# Optimizing Cryo-Focused Pyrolysis GC/MS for Tracing Soil Organic Matter Across Diverse Ecosystems

**DOI:** 10.1021/acs.est.5c16415

**Published:** 2026-03-16

**Authors:** Abrar Shahriar, Mavrik Zavarin, Erin E. Nuccio, Katherine E. Grant, Karis J. McFarlane, Jennifer Pett-Ridge, Daniel Toews, Jason P. Sexton, Rebecca Ryals, Joy Baccei, Aiden J. Berndt, Rene Boiteau, Ishtiaq Ahmed Jawad, Yu Yang, Sheel Bansal, Edward J. O’Loughlin, Kenneth M. Kemner, Roser Matamala, Keith D. Morrison

**Affiliations:** † Physical and Life Sciences Directorate, 4578 Lawrence Livermore National Laboratory, Livermore, California 94550, United States; ‡ Department of Life and Environmental Sciences, 33244 University of California, Merced, Merced, California 95343, United States; § Innovative Genomics Institute, University of California, Berkeley, Berkeley, California 94720, United States; ∥ University of California Natural Reserve System, Merced, California 95343, United States; ⊥ College of Science and Engineering, 5635 University of Minnesota, Minneapolis, Minnesota 55455, United States; # Department of Civil and Environmental Engineering, 6851 University of Nevada, Reno, Reno, Nevada 89557, United States; ¶ 95313 U.S. Geological Survey, Northern Prairie Wildlife Research Center, Jamestown, North Dakota 58401, United States; ∇ Biosciences Division, 1291 Argonne National Laboratory, Lemont, Illinois 60439, United States; ○ Environmental Science Division, Argonne National Laboratory, Lemont, Illinois 60439, United States

**Keywords:** soil organic matter, pyrolysis gas chromatography mass spectrometry, evolved gas analysis, organic matter characterization, molecular networking analysis

## Abstract

The cycling of organic matter in terrestrial soils and sediments is central to a range of biogeochemical processes that regulate nutrient cycling, crop productivity, trace gas emissions, and contaminant transport. Pyrolysis-gas chromatography/mass spectrometry (py-GC/MS) is a powerful tool for characterizing bulk soil organic matter (SOM) at the molecular level. In this study, we used a cryo-focused py-GC/MS system to analyze soil samples from seven diverse ecosystems: vernal pool, prairie pothole, temperate forest, tropical forest, tundra, wildfire-affected boreal forest, and grassland. We addressed a key bottleneck in molecular-level SOM characterization by developing an automated data analysis pipeline to optimize py-GC/MS and complementary evolved gas analysis/mass spectrometry (EGA/MS) methods, incorporating advanced tools for peak deconvolution, developing a custom compound class library, and implementing fragmentation spectrum-based molecular networking for the first time. This improved workflow was applied to soil samples from all seven ecosystems, including multiple depths and density fractions. Our findings demonstrate that ecosystem type plays a dominant role in shaping compositional differences in SOM. We also identified trends in the source of SOM compounds (e.g., microbial vs plant-derived) across soil depth and density fractions, which are critical for understanding persistence and turnover of SOM. Our molecular networking analysis indicated that although many compounds are widespread across ecosystems, others are restricted to specific environments, such as wetlands. This underscores the utility of molecular-level data in elucidating the complexity of SOM composition and the environmental drivers that shape it. Such molecular-level insights can deepen our knowledge of biogeochemical SOM cycles.

## Graphical abstract

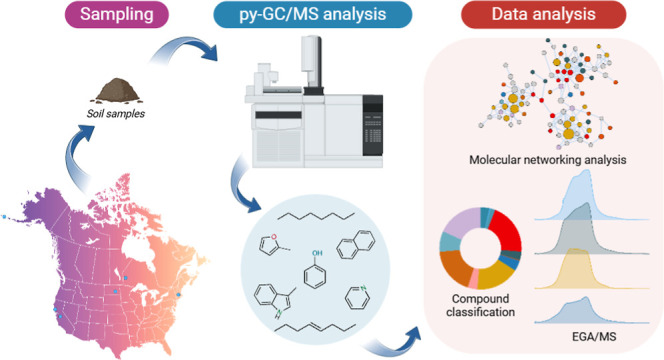

## 1 Introduction

Soil organic matter (SOM) contains the largest reservoir of organic carbon (OC) in terrestrial ecosystems (approximately 2400 Gt C, about three times higher than atmospheric C), and even a small increase in the release of this OC as carbon dioxide (CO_2_) or methane (CH_4_) could significantly impact atmospheric C levels^1–3^ SOM also plays a vital role in water-holding capacity, nutrient cycling, pollutant transport, and broader biogeochemical processes that sustain ecosystem functions.^4^ However, relating the chemical composition of SOM to its environmental function remains a formidable challenge because SOM is comprised of a complex mixture of molecules originating from a wide range of sources and exhibits diverse reactivities.^5,6^ Determining SOM composition and associated processes that govern SOM residence time and decomposition is necessary to predict changes in SOM inventories.

There is no current consensus on the extent to which molecular diversity, intrinsic chemical properties, and ecosystem properties governs the persistence of SOM. The long-held belief that stable SOM is formed from more recalcitrant fractions of polymerized OM has been challenged, and SOM is now thought to be made up of a continuum of plant and microbial biopolymers that are decomposed at different rates depending on their physicochemical environment.^2,7–12^ Some studies suggest that higher molecular diversity within SOM may confer resistance to decomposition, as it demands greater enzymatic versatility and energy investment from microbial communities.^1,13^ In contrast, Jones et al.^14^ reported that more persistent SOM, with an older radiocarbon age, had lower molecular diversity and a greater abundance of aromatic compounds relative to fresh SOM. Identifying the sources of soil OC is also critical for understanding C dynamics in soil, as both plant- and microbe-derived compounds can contribute to the persistent SOM pool to varying degrees through different physical and chemical mechanisms.^15–17^ Analyses that can accurately characterize SOM and address the ongoing debates surrounding the relationship between SOM composition and persistence are urgently needed.

A wide variety of analytical techniques have been employed to characterize SOM including high-resolution mass spectrometry (HRMS), infrared spectroscopy (IR), nuclear magnetic resonance (NMR), synchrotron-based mass spectrometry and many other emerging chemical imaging techniques.^18–22^ Each of these techniques has strengths and inherent limitations.^22,23^ Most liquid chromatography (LC) or direct injection HRMS analyses require soil extraction procedures, which may not capture the full compositional diversity of SOM.^18^ In contrast, IR and NMR analyses are limited to functional group characterization and do not resolve molecular formulas or structures of SOM compounds. Pyrolysis-gas chromatography/mass spectrometry (py-GC/MS) offers a valuable alternative that overcomes many of these limitations, providing detailed molecular information on whole soil samples with minimal sample preparation. However, the major bottleneck limiting the widespread use of py-GC/MS lies in data analysis, which often relies on manual peak curation, lacks high-confidence compound annotations with molecular structure information, and depends on applications with limited user-friendly interfaces.^24–28^ Evolved gas analysis/mass spectrometry (EGA/MS) is a complementary technique to py-GC/MS, capable of providing thermal profiles of all SOM compounds collectively across a wide temperature range. The combination of these techniques can provide a detailed picture of SOM characteristics, as py-GC/MS reveals its overall molecular composition, while EGA/MS offers insights into the thermal stability of the associated compounds.^29^ Nevertheless, the combined application of py-GC/MS and EGA/MS remains limited, and only a few studies have used this approach for complex environmental samples, leaving much of its potential understudied.^25,30,31^ Thus, there is a need to improve the entire workflow–from implementing cryogenic trapping to enhance compound capture during analysis, to developing automated, user-friendly data processing pipelines capable of handling the complexity of the resulting data sets.^32^

For this study, we developed a novel automated workflow to derive SOM characteristics from py-GC/MS data and tested this approach by applying it to a diverse set of ecosystem soils. We optimized py-GC/MS and EGA/MS analyses by integrating advanced tools for deconvoluting complex py-GC/MS data, applying an expanded mass spectral library, and developing a custom library to assign compounds to designated classes. Fragmentation spectrum based molecular networking analysis was performed on the py-GC/MS data for the first time and bin annotated features into compound classes related to biological origin. We then applied these improved techniques to soil samples collected from a wide range of ecosystems across different soil depths, and density fractions. Overall, our findings provide an accessible and sophisticated method for characterizing SOM in diverse environmental settings, enabling future research into the controlling factors and processes of SOM dynamics in natural ecosystems.

## 2 Materials and Methods

### 2.1 Soil Sampling and Preparation

Soil samples were collected from diverse ecosystems across the United States and Canada, including vernal pools in California, prairie potholes in North Dakota, temperate forests in Massachusetts, tropical forests in Puerto Rico, boreal forests in Ontario, tundra in Alaska, and Mediterranean grasslands in California (Figure 1). A detailed description of the soil collection, site description, storage, preparation, and general properties of the soil samples can be found in the Supporting Information section (Text S1 and Table S1). For the py-GC/MS and EGA/MS analysis, dry bulk soil samples were used. A subset of soil samples was fractionated into two density fractions–heavy fractions (HF) and light fractions (LF).^33^ HF and LF mainly refer to mineral-associated organic matter (MAOM) and particulate organic matter (POM), respectively, although their interpretation can be limited by incomplete dispersion of fine particles and the difficulty of separating very fine particles with different mineralogy.^34^ The detailed density fractionation procedure can be found in Supporting Information (Text S2). Elemental analysis of C and N in a subset of soil samples was carried out using Vario El Cube (Elementar, Germany). Organic standards (kraft lignin, humic acid, and l-tryptophan) were also used in this study; detailed information is provided in the Supporting Information (Text S3).

**Figure 1:**
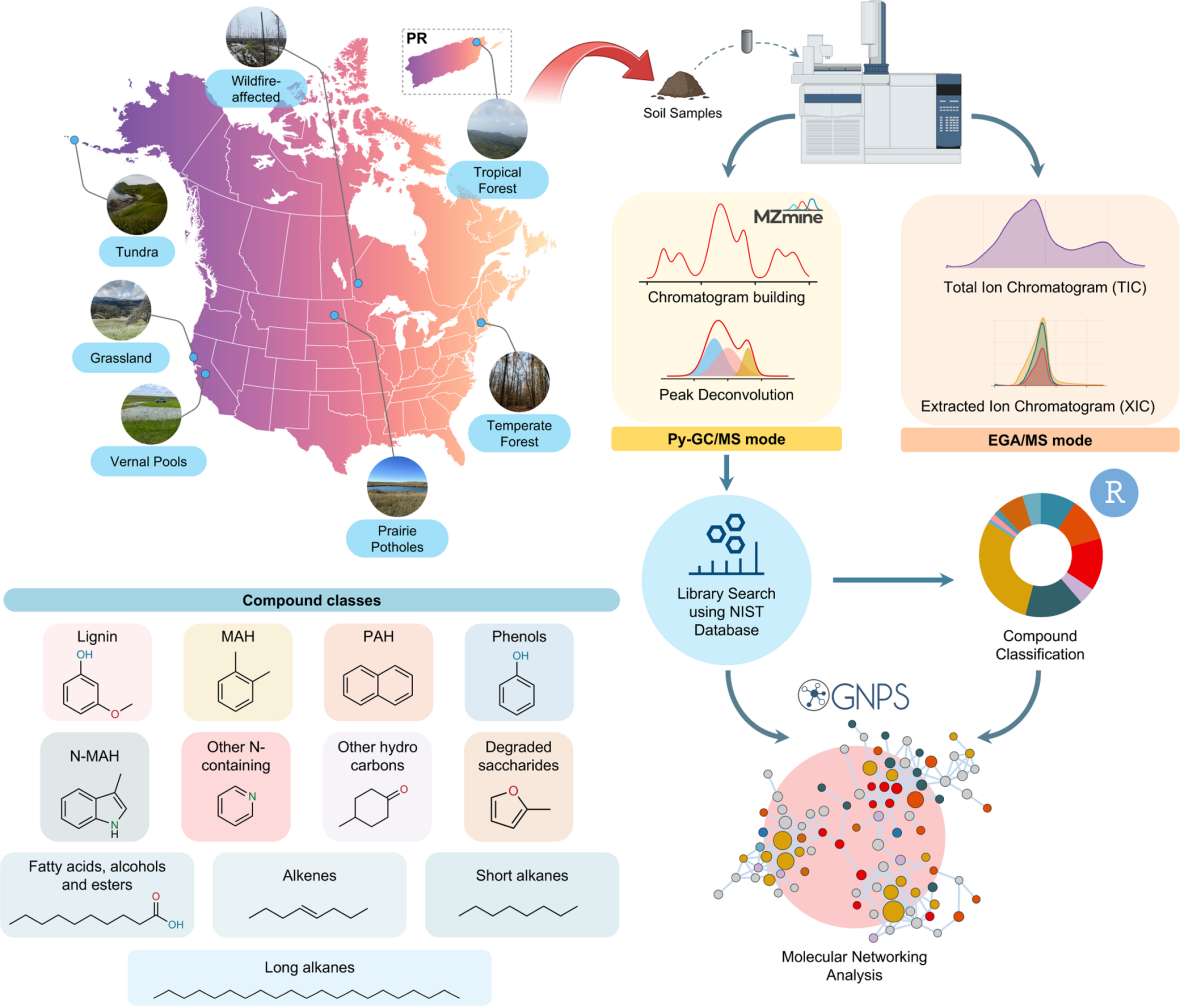
Overall soil sampling location and workflow of pyrolysis-gas chromatography/mass spectrometry (py-GC/MS) and evolved gas analysis/mass spectrometry (EGA/MS) analysis. Compound classes with representative example compounds identified through library searches using the National Institute of Standards and Technology (NIST) database.

### 2.2 Analytical Methods

#### 2.2.1 Single Shot Py-GC/MS Analysis

Py-GC/MS analyses were carried out using a Frontier multishot pyrolyzer (EGA/PY-3030D, Frontier Laboratories, Fukushima, Japan) equipped with autoshot sampler (AS-2020E), selective sampler (SS-2010E) and microjet cryo-trap (MJT-1035E) in cryo-focused analysis mode. The pyrolyzer system was coupled with an Agilent 7890B GC and Agilent 7010B mass spectrometer (Agilent Technologies, Santa Clara, CA, USA). Soil samples were introduced into the instrument in Frontier Eco-Cups sealing the top of the cups with sterilized quartz wool. The quartz wool was sterilized in a tube furnace at 1500 °C for 15 s to remove any organic impurities. Samples underwent pyrolysis at 500 °C in most cases, unless otherwise indicated, to avoid overpyrolysis that can occur at higher temperatures. The pyrolyzed products were cryo-focused at −180 °C for 3 min. Helium (He) was used as carrier gas while N_2_ was used in the collision cell of the mass spectrometer. For chromatographic separation an Agilent HP-5MS-UI (30 m × 250 μm inside diameter × 0.25 μm inner coating; (5%-phenyl)-methylpolysiloxane phase capillary column) column was used. The GC oven was initially held at 40 °C for 5 min, then ramped at 2 °C/min to 200 °C, where it was maintained for an additional 5 min, before ramping up at 20 °C/min to 320 °C. The inlet to the GC–MS was kept at 300 °C and the septum purge flow was 3 mL/min. The inlet was run in split mode, with the gases entering the column at a split flow of 15:1 (He: sample gas). The mass spectrometer was set to scan on the first quadrupole mass spectrometer from *m*/*z* 29 to 850, using a 300 ms scan time. A subset of samples was also analyzed using an Agilent DB-Wax-UI column (30 m × 250 μm inside diameter × 0.25 μm inner coating; polyethylene glycol (PEG) column) for method development purposes (detailed information about the parameters and columns used can be found in Supporting Information, Text S4).

#### 2.2.2 EGA/MS Analysis

EGA/MS analysis was carried out using the same instrumental setup except for the GC column. An EGA steel column (3 m × 150 μm inside diameter) with no inner coating was used to capture evolved gases with no chromatographic separation. Samples were measured in ramped heating mode starting at 100 °C (1 min hold) and then heated at a rate of 10 °C/min to 900 °C. The GC oven was kept at 300 °C during analysis. Similar to py-GC/MS analysis, He was used as the carrier gas while the collision cell in the mass spectrometer used N_2_ gas. Helium pressures on the EGA column were set at 16.95 psi in constant pressure mode. The inlet to the GC/MS was kept at 300 °C and the septum purge flow was 3 mL/min. The inlet was run in split mode, with the gases entering the EGA column at a split flow of 15:1 (He: sample gas). The mass spectrometer was set to scan on the first quadrupole mass spectrometer from mass 29 to 650, using a 2000 ms scan time.

### 2.3 Data Analysis

#### 2.3.1 Processing of Py-GC/MS Data

All the data generated from py-GC/MS analysis were converted by MSconvert (ProteoWizard) to mzML format for subsequent data processing by MZmine3 (version 3.9.0, http://mzmine.github.io/). The intensity threshold was set to 10^5^ and the Automated Data Analysis Pipeline (ADAP) chromatogram builder and resolver was used to build initial chromatograms.^35^ The signal-to-noise (S/N) was set at ≥ 5 and the minimum feature height was set at 10^5^. Generated GC chromatograms were deconvoluted using the multivariate curve resolution algorithm where retention time (RT) toleration was set to be 0.05 min. All the deconvoluted features (duplicates in case of single samples, or combination of different samples) were combined using ADAP GC aligner algorithms with a threshold score of 0.7. Combined deconvoluted features were annotated using the National Institute of Standards and Technology (NIST) library database (2023 Edition) and NIST MS search 3.0 embedded in MZmine3. Minimum cosine similarity values were set at 0.7 unless otherwise stated. All the results were exported as csv files for further data analysis. For subsequent networking analysis, additional quantification table and mgf files were exported.

The annotated features were classified into 12 distinct compound classes based on their potential origin and chemical structure similarity (Supporting Information, Table S2 and Text S5). The classes are alkenes, degraded saccharides, fatty acids, alcohols and esters, lignin, long alkanes, monocyclic aromatic hydrocarbons (MAH), nitrogen containing monocyclic aromatic hydrocarbons (N-MAH), other hydrocarbons, other N-containing compounds, phenols, polycyclic aromatic hydrocarbons (PAH), and short alkanes.^25,26,28^ We built a library based on >1600 unique annotated compounds found in all our analyzed samples, along with their respective classes (Supporting Information, Table S2 and Compound classes library data file). The library is not limited to the compounds currently included and can be expanded as additional unclassified compounds are identified in future studies. We developed an in-house R script to automatically classify annotated features from each sample based on our custom compound library with predefined classes.^36^ The library file can be found as a spreadsheet in the Supporting Information. The relative abundance of annotated features was presented based on integrated peak heights. The relative abundance of different classes based on peak heights does not represent the carbon mass of each class.

#### 2.3.2 EGA/MS Data Analysis

EGA/MS data files were analyzed using Agilent MassHunter Qualitative Analysis 10.0 (Agilent Technologies Inc., Santa Clara, CA, USA) software. Extracted ion chromatograms (XIC) were built for each compound class combining major *m*/*z* peaks of identified compounds. Mass spectra were also extracted from the EGA curve using MassHunter.

#### 2.3.3 Molecular Networking Analysis

Molecular networking analysis based on the deconvoluted EI GC/MS data was performed using Global Natural Product Social (GNPS) platform.^37^ Clusters in the overall network were created in GNPS based on the spectral similarity of the preprocessed deconvoluted fragmentation spectrum using MZmine3.^38^ The minimum pair cosine similarity threshold for edges was set to 0.7, and the network TopK parameter (maximum number of neighboring nodes per node) was set to 10. Cytoscape (version 3.9.1) was used to visualize molecular networks.^39^ The input files used in our molecular networking analysis did not contain any compound class data which were added separately after exporting the networks from GNPS using Cytoscape. Molecular networking analysis was not used to identify any additional compounds beyond those detected through the NIST library database search. All compounds shown in the molecular networking results were identified based on the NIST library search, as described in Section 2.3.1. We wrote a separate python script to further classify the unannotated nodes based on the cosine similarity among nodes in clusters. Additional details are provided in the Supporting Information (Text S6).

### 2.4 Statistical Analysis

All the statistical analyses were done using R version 4.5.1 in RStudio.^40^ Shannon, and Simpson diversity indices were used to show the diversity of SOM composition based on the pyrolysates. Details of the indices are in the Supporting Information (Text S7). We performed hierarchical clustering of log-transformed compound abundances and visualized the resulting patterns as a heatmap using the *pheatmap* R package.^41^ To assess differences in overall compound composition among soil samples, we computed Bray–Curtis dissimilarities from replicate-averaged compound intensities (*vegdist*, *vegan*) and tested for group separation using PERMANOVA (adonis2, 999 permutations).^42^ Correlation between different ecosystem SOM compounds were calculated using Pearson’s correlation with a significance value of *p* < 0.001.

## 3 Results and Discussion

### 3.1 Method Development

We evaluated multiple factors to optimize both the analytical (sample concentration, column selection) and data processing (peak deconvolution, compound identification, and class assignment) workflows, ensuring their applicability not only to our soil samples but also to a broad range of environmental materials. Although our py-GC/MS setup included double-shot capabilities, which allow for thermal desorption in the first shot and pyrolysis in the second, we focused solely on pyrolyzing the samples.^43^ This protocol was supported by Lyu et al.,^25^ who reported that only 2.7 ± 1.9% of the compounds in their samples were thermally desorbed using the double-shot method. Furthermore, as shown later in this study, EGA/MS analysis supported the use of pyrolysis mode at 500 °C.

To evaluate the performance of compound detection, we tested two different columns: DB-Wax (polar stationary phase) and HP-5MS (nonpolar stationary phase). Overall, the HP-5MS column captured a greater number of compounds based on the analysis of vernal pool samples, detecting up to five times more unique annotated compounds than the DB-Wax column (Supporting Information, Figure S1). This is likely due to greater compound retention at the lower temperature range for the wax-based columns (250 °C) compared to the siloxane-based columns (325 °C). Based on this performance, only the HP-5MS column was used for further analyses. We also tested optimal sample loading and determined that 200 to 300 μgC per sample produced strong SOM signals with a clean baseline. For example, in the vernal pool upland sample collected from 10–30 cm depth, the number of unique classified compounds identified from 108, 295, and 444 μgC samples was 99, 158, and 174, respectively. However, samples exceeding 400 μgC showed increased column bleed and poor baseline quality (Supporting Information, Figure S2A). A sample of over 1 mgC produced a noisy chromatogram with a poor baseline (Supporting Information, Figure S2B), making deconvolution and data processing more difficult. Considering all factors, we used 200 to 300 μgC per sample for the majority of our analyses. Our analytical pipeline demonstrates improved efficiency and compound identification rates relative to AMDIS, a commonly used tool for processing py-GC/MS data.^44^ One major limitation of AMDIS is its lack of support for large scale batch processing and limited scalability when handling large data sets.^38^ MZmine, utilizing the ADAP spectral deconvolution workflow, addresses these challenges and enhances analytical outcomes by offering customizable parameters, integration with the NIST database for direct compound identification, and the ability to generate the required input files for molecular networking analysis in the GNPS environment.^38,45,46^

To validate our molecular classification approach for distinguishing OM with distinct compositions, we also analyzed representative materials, including kraft lignin, humic acid, and selected protein monomer. Analysis of the kraft lignin standard revealed that approximately 90% of the classified compounds fell into three of our designated classes: lignin (50.5 ± 3%), phenols (24.5 ± 2%), and MAHs (13.4 ± 0.6%) (Supporting Information, Figure S3). These observations are consistent with the structural characteristics of lignin, which are expected to produce pyrolysates such as syringol, guaiacol, and related compounds, along with MAHs and phenols generated through depolymerization and demethoxylation during pyrolysis.^47–49^ Aliphatic and N-containing compounds, however, should be absent or minimal in lignin-derived pyrolysates. In contrast, the humic acid standard had a far more complex profile, with compounds distributed across a broader range of classes, including a high abundance of degraded saccharides (39.3 ± 0.8%) and phenols (19.3 ± 3.4%). Aliphatic classes, such as short alkanes (0.8 ± 0.1%) was present in small amounts in the humic acid but completely absent in the lignin standard. Humic substances may yield compounds from a wider array of compound classes depending on their origin and complexity.^50^ The α-amino acid l-tryptophan produced pyrolysates that were primarily classified as N-MAH compounds (e.g., indole and β-carboline derivatives) and other N-containing compounds (e.g., carbamic acid). The formation of these pyrolysates can be attributed to decarboxylation, dehydrogenation, and demethylation reactions occurring during pyrolysis.^51^ Although α-amino acid monomer standards do not capture the complexity of environmental samples, their pyrolysates can provide valuable insight into compound origins. Additionally, these standards confirm that the pyrolysis process does not produce artifacts, defined as products inconsistent with the expected chemical structure of the precursor, thus validating the observed compound assignments. The standards served to confirm our classification framework, support comparisons with prior studies, and distinguish the sources of specific pyrolysates. The classes we defined can serve as a flexible framework, and the classification scheme can be adjusted to accommodate simpler or more detailed classification depending on the objectives of future analyses.

### 3.2 Composition, Diversity, and Sources of SOM Compounds Across Ecosystems

We observed a large variation in the abundance of compound classes among soil samples collected from multiple ecosystems. For all soil samples, we annotated 50.6 ± 10.9% of the identified peaks using the NIST database and classified them into 12 compound classes. In all ecosystems, MAH was the largest contributor to overall SOM composition, ranging from 45.9 ± 3.3% in wildfire-affected boreal forest soil to 31.4 ± 1.4% in tropical forest soil. The second-largest contributing compound class was ecosystem-dependent (Figure 2A). N-MAH compounds were most abundant in tundra soils (16.3 ± 1.5%), while other N-containing compounds abundance was higher in tropical forest soil (21.5 ± 4.8%). High N-containing compounds in tropical forest, such as those in Puerto Rico, result from open N cycle and N-rich soils.^52,53^ The two wetland ecosystems, vernal pools and prairie potholes, showed similar proportions across multiple compound classes, with the main difference observed in the phenol class (3.1% vs 0.6%, respectively). Wildfire-affected soils from the boreal forest also had a higher abundance of the lignin class (9.1 ± 1.5%) compared to other soils. Based on hierarchical clustering, tundra and temperate forest soils grouped together, prairie potholes clustered with vernal pools, and grasslands and tropical forests formed a separate cluster (Supporting Information, Figure S4). Wildfire-affected boreal forest soils were distinguished by unique compound enrichments not observed in other ecosystems, suggesting disturbance-driven chemical shifts. These clustering patterns aligned with the PERMANOVA results (pseudo-*F* = 11.18, *R* ^2^ = 0.91, *p* = 0.001), confirming that ecosystem type could be a major factor driving compositional differences. However, a more extensive sample set from each ecosystem is required to fully elucidate differences in SOM composition among ecosystems.

**Figure 2:**
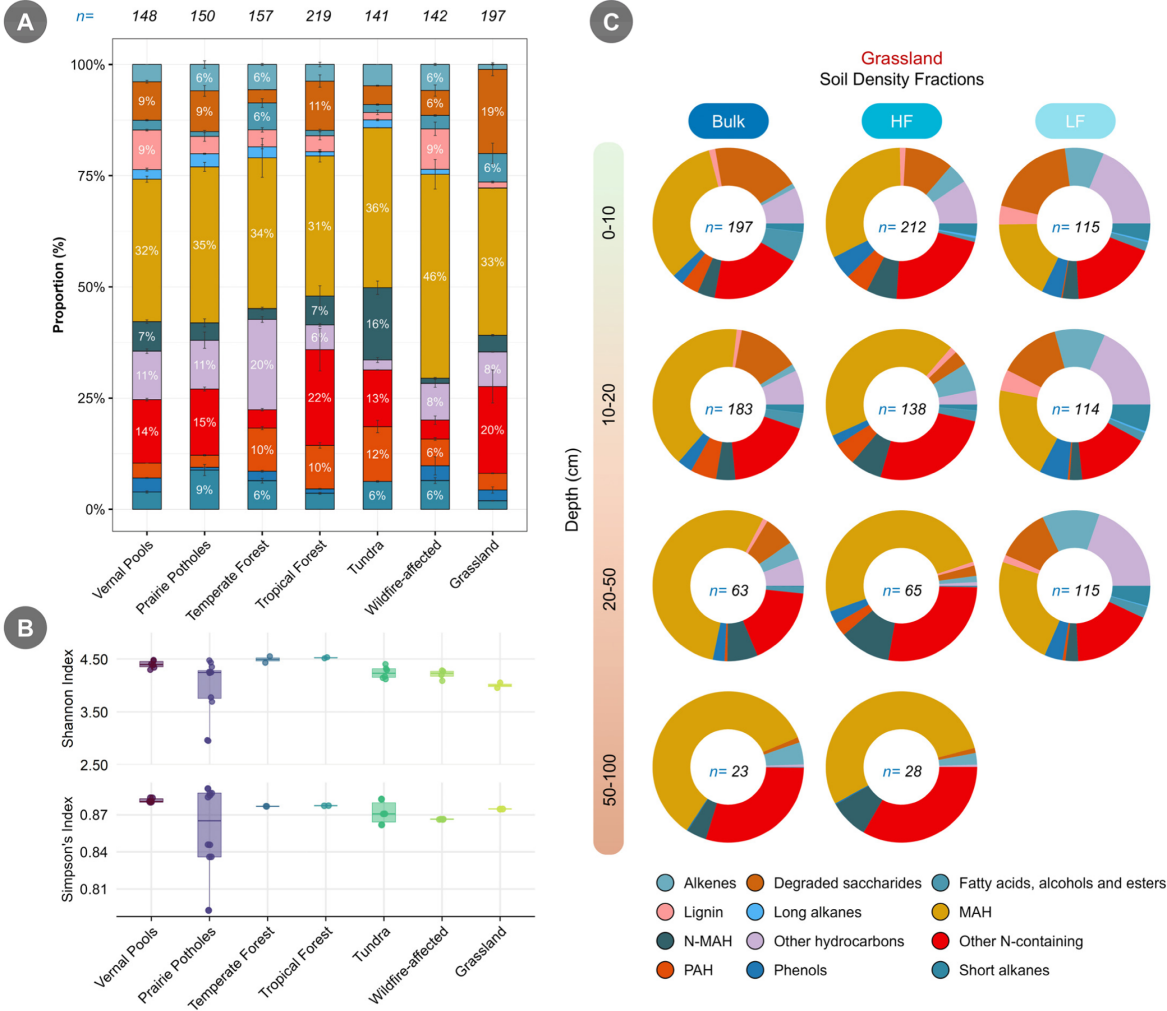
(A) Relative abundance of different compound classes in various ecosystems soil samples. The results are for bulk topsoil. Results from vernal pool, prairie pothole, and tundra soil are for upland 0–10 cm depth, T3 site, and SC1 site, respectively (description of sites are in SI). Error bars represent one standard deviation. *n* represents the average number of unique compounds that were found per ecosystem sample. The legends for panels A and C are the same. (B) Calculated diversity indices based on the full set of bulk topsoil samples from each ecosystem. Error bars represent one standard deviation. (C) Distribution of compound classes in bulk soil, and in the heavy (HF) and light (LF) fractions of grassland soil at different depth profiles. The value of *n* inside each donut chart indicates the average number of unique compounds identified per sample.

Understanding SOM diversity is important, as it may be linked to stability.^54^ Microbial decomposition and the subsequent transformation of SOM can be regulated by its chemical diversity.^1,55^ Our enhanced py-GC/MS workflow enables high-confidence annotation of up to 219 unique compounds within a single sample, yielding a more detailed understanding of SOM diversity compared to previous py-GC/MS studies.^14,25,28,56–59^ Both Shannon and Simpson diversity indices indicated that soils from vernal pools, tropical forests, and temperate forests exhibited the highest diversity (Figure 2B). In contrast, wildfire-affected boreal forest soils had on average the lowest diversity according to the Simpson’s index (0.87), whereas grassland soils had the lowest diversity based on the Shannon index (4). Davenport et al.^13^ also demonstrated that ecosystem type strongly influences molecular diversity, with tundra and grassland soils showing reduced diversity in tandem LC–MS analyses, a pattern consistent with our observations. Prairie pothole soil samples displayed substantial differences in terms of identified compounds from each sampling site. These samples were collected from two hydrological types: permanently inundated wetlands (P sites) and seasonally inundated wetlands (T sites). No clear differences were detected between the two sites and hierarchical clustering using Bray–Curtis dissimilarities indicated substantial compositional overlap between P and T site samples (dissimilarity = 0.65) (Supporting Information, Figure S5). Nevertheless, variations in overall compound class distributions among samples from the same ecosystems in prairie potholes and tundra (Supporting Information, Figures S5 and S6, respectively) underscore the need for more detailed investigations into the environmental factors that influence SOM composition, even within a single ecosystem type.

We also observed substantial variation in SOM compound class composition across depth profiles and soil density fractions within a single ecosystem. Topsoil (0–10 cm) and subsoil (10–30 cm) samples collected from vernal pools showed that, with increasing depth, both N-containing compound classes increased, while SOM compounds belonging to classes such as lignin and phenol decreased consistently across all three sampling zones (i.e., upland, transition, and center of wetland) (Supporting Information, Figure S7). The pattern might arise from increased plant inputs in the topsoil, whereas subsoil SOM is more strongly shaped by microbial contributions.^13,14^ The clustering analysis indicated clear depth-dependent differences; while sampling zones (among three zones of vernal pools at the same depth) showed no significant variation (PERMANOVA, pseudo-*F* = 0.47, *R* ^2^ = 0.24, *p* = 0.49). Diversity decreased from topsoil (mean Shannon index = 4.56 ± 0.09) to subsoil (3.80 ± 0.47), consistent with the trend observed by Jones et al.^14^ (Supporting Information, Figure S7).

Although accurate assignment for the origin of compounds across classes is challenging, reasonable assumptions can be made based on their structural characteristics. For example, lignin-derived compounds are primarily associated with plant material.^60,61^ Similarly, long-chain alkanes and unsaturated hydrocarbons such as alkenes are thought to be largely plant-derived, although microbial contributions cannot be entirely ruled out.^28,62^ In contrast, most N-containing (both N-MAH and other N-containing) compounds are typically linked to microbially processed SOM.^25,28,63^ Beyond these five classes, distinguishing origin becomes more challenging, as many compounds can be derived from both microbial and plant sources. For instance, phenolic compounds may originate from plant lignin, as demonstrated earlier in this study, or be produced by microbial processes.^28,64^ Stable isotope–based analyses can provide insights into the relative contributions of different sources to the SOM pool.^65,66^ Even when the five classes are considered separately, it was evident that the contribution of microbially derived SOM class abundance increased with depth in the HF, whereas it remained much lower in the LF. Based on our standards, the other hydrocarbons and phenolic compounds in the LF most likely originate from plants rather than microbial sources. In grassland soil, the abundance of different classes, especially aliphatic compound classes (i.e., alkene, short, and long alkanes), decreased with depth (Figure 2C). The number of annotated features also decreased with depth and at 50–100 cm depth the bulk and HF soil samples chiefly consisted of MAH, N-MAH, and other N-containing compounds. For example, the abundance of MAH increased gradually from 32.1% to 53.9% in HF fraction from 0 to 10 cm depth to 50–100 cm depth. Radiocarbon analysis of this grassland soil showed that Δ^14^C values for bulk soil and density-fractionated samples became increasingly depleted with depth.^33^ For example, Δ^14^C values decreased from 31 ± 3‰ at 0–10 cm to −468 ± 3‰ at 50–100 cm. However, the LF showed no drastic change over the overall composition of different classes over the depth gradient. The number of identified compounds across depth also remained the same.

Many studies have shown that the stable pool of SOM in the HF (which primarily represents MAOM) is predominantly composed of microbially processed SOM, accounting for more than 50% of total soil OC in grassland and temperate agricultural soils.^15,67–69^ However, Chang et al.^70^ challenged this view, reporting that microbial input accounted for less than 50% based on their stoichiometric approach. Additionally, they observed that the microbial contribution increased with depth in grassland soils, from a mean value of 24% at depths <20 cm to 34% at depths >20 cm. From our observations, the microbial contribution accounted for an average of 23.2% at 0–10 cm depth and increased to 33.8% at 50–100 cm depth in grassland bulk soil samples, when considering N-MAH, and other N-containing compounds as primarily of microbial origin. However, the total number of identified compounds at 50–100 cm was very limited.

### 3.3 Thermal Stability of SOM Using EGA/MS Analysis

We employed EGA/MS analysis to assess the thermal stability of SOM using the EGA curves and the corresponding MS spectra at each temperature point. EGA curves showed differences in the composition of thermally evolved organic compounds across ecosystems (Figure 3A). For the same amount of total C (around 250 μg per sample), the abundance of SOM-derived compounds varied, indirectly suggesting compositional differences among ecosystem samples. The major peaks in the EGA results indicate that ∼500 °C is a suitable temperature for py-GC/MS analysis across the diverse samples analyzed and avoids alterations of pyrolysates that can occur at elevated temperatures (Figure 3A).^71^ Soil from temperate forest and vernal pool yielded the largest fractions of evolved SOM compounds among the investigated samples, as evident from their MS spectra at the highest peak temperature (Supporting Information, Figure S8).

**Figure 3:**
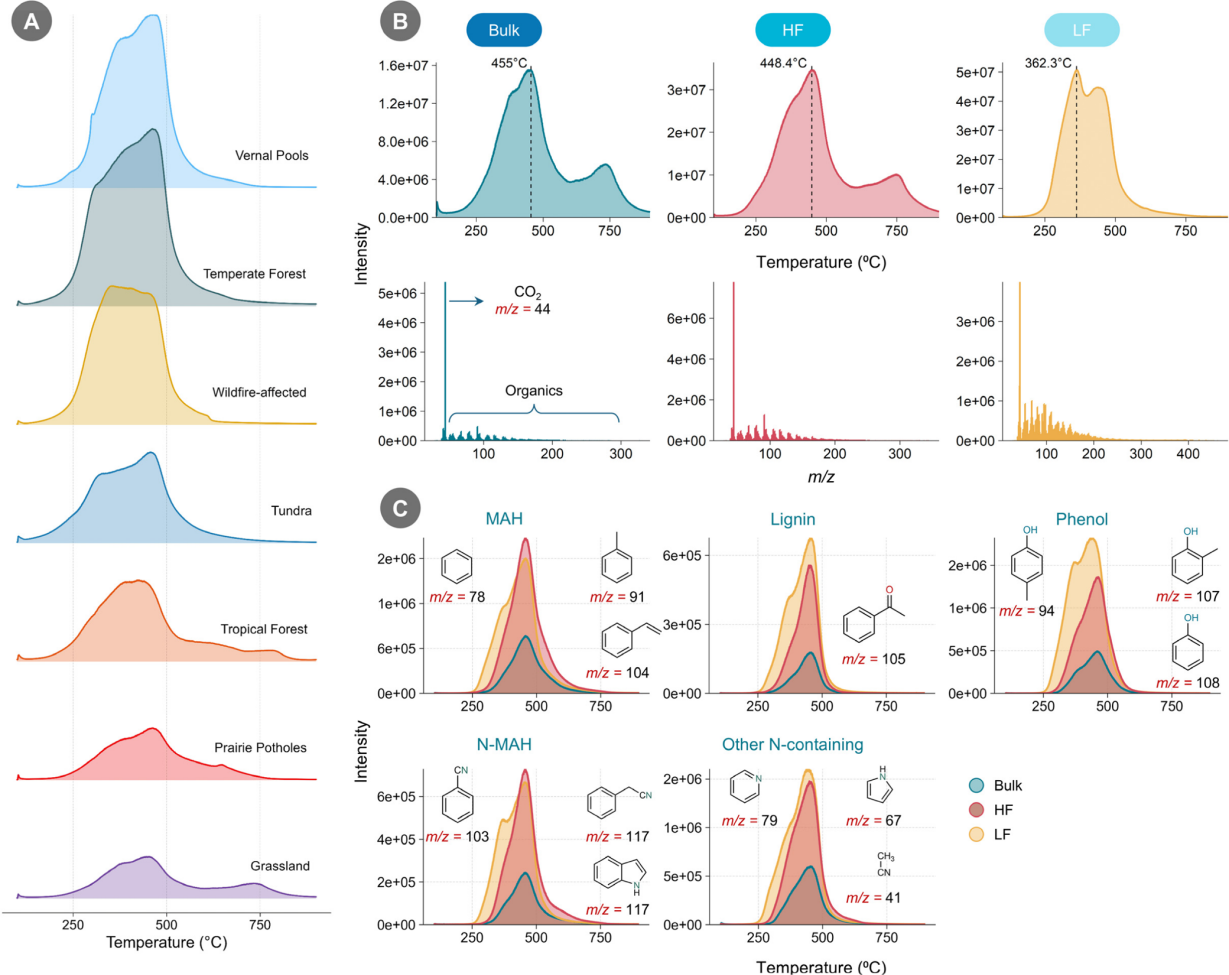
(A) Evolved gas analysis (EGA) curves of soil samples from different ecosystems (same samples as shown for Figure 2). The *y*-axis (not shown in the figure) represents the intensity on the same scale for each panel. (B) EGA curves of bulk, heavy (HF), and light (LF) soil fractions from grassland (0–10 cm depth). The temperature indicated in each panel represents the temperature at which signal intensity was highest. The corresponding mass spectra (MS) for each EGA panel is shown at that temperature. (C) Extracted ion chromatograms (XICs) of five different compound classes. The *m*/*z* values shown represent the most abundant *m*/*z* for each compound class. Except for lignin, XICs for the other classes were generated by merging multiple *m*/*z* values into a single chromatogram.

In some soils, particularly those from the grassland, an additional peak was observed around 730 °C. Peaks above 650 °C may indicate the presence of inorganic carbon (IC) or highly stable MAOM and tar.^30,72^ Tundra soil, which exhibited the highest abundance of PAHs among the soils analyzed, also showed the presence of naphthalene (*m*/*z* = 128) at 650 °C, indicating that PAHs required elevated temperatures to thermally evolve (Supporting Information, Figure S9). However, our extended cryo-focusing method (3 min duration) still captured these compounds. The humic acid standard and pure CaCO_3_ showed peak temperatures around 476.6 and 723.2 °C, respectively, reflecting the general temperature ranges for OC and IC (Supporting Information, Figure S10). The MS spectra of prairie pothole soil at 700.2 °C primarily consisted of a CO_2_ peak (*m*/*z* = 44), suggesting a higher proportion of IC, which was supported by analysis of acid-treated samples as the major peak at >700 °C was completely gone (Supporting Information, Figure S11A). Grassland and tropical soils showed peaks at around 730 and 780 °C, respectively. However, tropical forest soil samples used in this study did not have any detectable IC.^73^ Similarly, the acid-treated grassland sample exhibited a peak at 780 °C but lacked the sharp decline observed in the CaCO_3_ standard or the IC–rich prairie pothole sample, suggesting the absence of IC (Supporting Information, Figure S11B). Thus, the high-temperature peaks in the grassland and tropical soil samples likely reflect the presence of thermally stable SOM. This thermally stable SOM might be more resistant to pyrolysis or associated with minerals. Furthermore, the presence of the high-temperature peak in bulk soil and HF, but its absence in LF, suggests more stable, mineral-associated SOM in the bulk soil.

The EGA curves of grassland bulk soil and HF showed comparable patterns, with both exhibiting their highest peaks at similar temperatures (455 and 448 °C, respectively). This likely reflects the high proportion (>80%) of HF in the grassland bulk soil.^33^ In contrast, LF displayed a distinct profile, with its highest peak occurring at a lower temperature (362 °C) and showing greater abundance of SOM, with an absolute intensity of 5.07 × 10^7^ (Figure 3B). The MS spectra at the highest peak temperatures for bulk, HF, and LF revealed that the CO_2_ peak was dominant in both bulk and HF, whereas LF displayed a range of higher-abundance organic compound peaks relative to CO_2_. The LF curve exhibited two prominent peaks at 362 and 436 °C, indicating a greater abundance of more “thermally labile” organic matter not observed as distinctly in HF.^30^ The XIC of CO_2_ showed two major peaks in both bulk and HF samples, while LF exhibited a gradual decrease in CO_2_ intensity over the temperature ramp (Supporting Information, Figure S12). XICs of major classes, based on the most abundant peaks from the most abundant compounds within each class, demonstrated clear differences in compound abundance among the three fractions. Lignin (represented by acetophenone, *m*/*z* = 105) and phenol (including phenol, 2-methylphenol, and p-cresol, *m*/*z* = 94, 107, and 108, respectively) were found in higher abundance in LF, suggesting their predominant plant origin (Figure 3C). In contrast, MAH and N-MAH were more abundant in HF, while other N-containing compounds were also found in higher amounts in LF. The highest peak temperature ranges were consistent across classes, for example, ranging from 448 to 463 °C in HF. XICs of representative compounds show class-specific thermal behavior across ecosystems and depths, these insights cannot be captured just by py-GC/MS alone.

### 3.4 Molecular Networking–Driven SOM Annotation and Ecosystem Linkages

To expand compound class assignment beyond our existing spectral libraries, we employed molecular networking. This approach is based on the principle that structurally similar molecules produce similar MS fragmentation spectra.^38^ By connecting compounds with high spectral similarity (cosine score >0.7), we constructed networks that reveal relationships between annotated and structurally similar unannotated molecules. The overall network contained 279 nodes excluding any singlet nodes and 453 edges (Figure 4A). The median pair cosine similarity was 0.89, and 416 edges exhibited pair cosine similarity values greater than 0.8 (Supporting Information, Figure S13).

**Figure 4:**
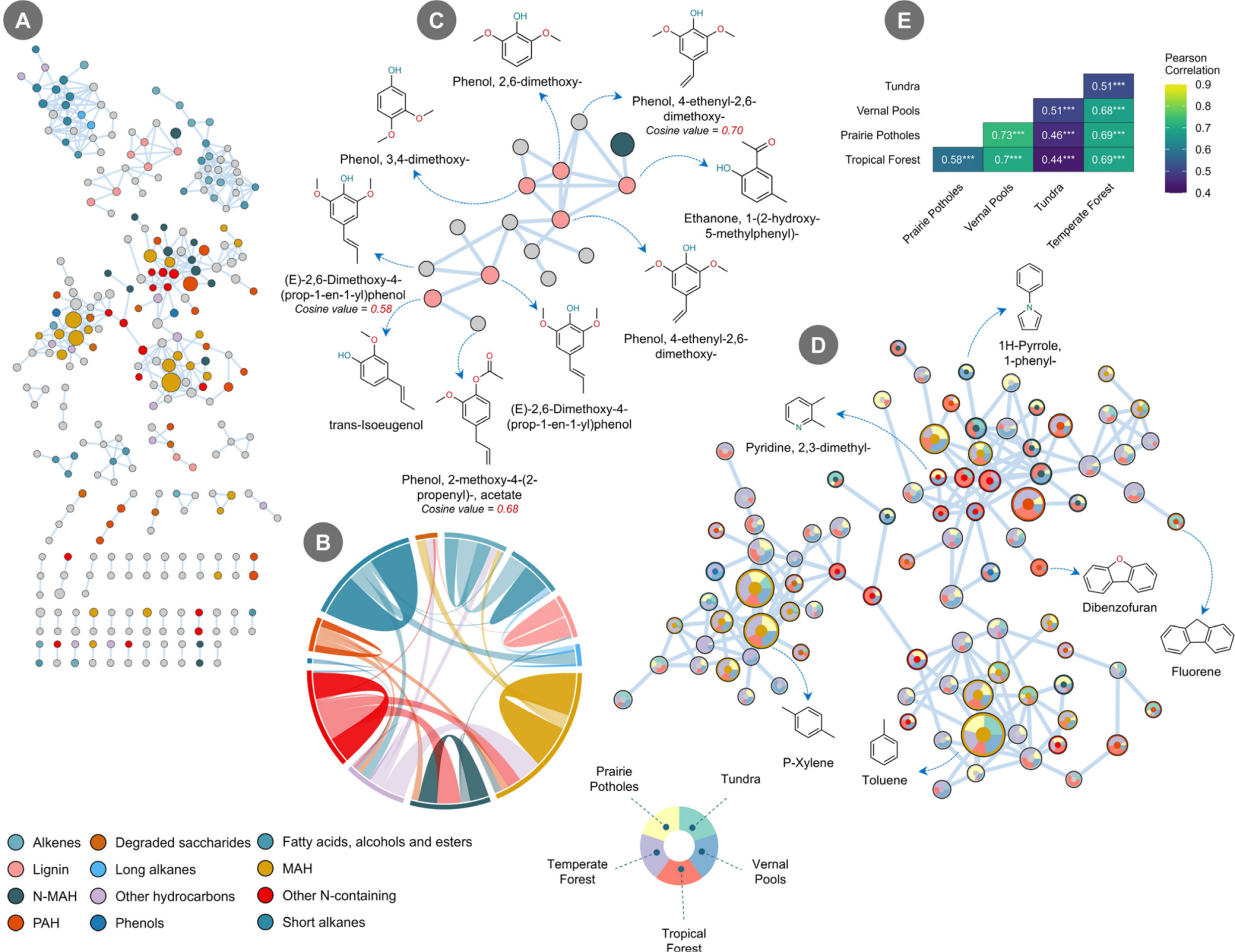
(A) Overall molecular networking analysis based on soil samples from five ecosystems: vernal pool, prairie pothole, temperate forest, tropical forest, and tundra. The size of the nodes in each cluster represents their relative abundance. Gray nodes indicate unclassified groups. The legend for compound classes is consistent across panels A–D. (B) Chord diagram showing connections between annotated nodes of the same or different compound classes. (C) A cluster primarily composed of lignin class compounds. (D) The largest cluster by total number of nodes, showing the relative abundance of each compound across different ecosystems. The color of each node represents the compound class, while the donut chart inside each node shows the relative abundance of that compound across five ecosystems. (E) Correlation matrix among soil samples from different ecosystems, based on the abundance of identified compounds. *** indicates *p* < 0.001.

Our analysis first confirmed that annotated compounds consistently clustered with other molecules belonging to the same chemical class. Among the 157 edges connecting two classified nodes, 80 occurred between nodes of the same class (Figure 4B). This indicates that clustering can reveal additional compounds. For instance, a cluster of classified lignin class compounds with high cosine similarity scores (>0.7) suggested that neighboring unclassified nodes are likely to also represent lignin-related compounds. When the cosine similarity threshold was lowered to better capture matches for unclassified compounds, additional lignin-derived molecules were identified. For example, 4-ethenyl-2,6-dimethoxy-phenol, initially unclassified due to a cosine score below 0.7, was connected to 2,6-dimethoxy-phenol, a known lignin compound with a similar MS fragmentation pattern (Figure 4C). A similar connection was observed for 2-methoxy-4-(2-propenyl)-phenol acetate.

Based on this validation, our Python workflow then used these clusters to systematically classify unannotated nodes by assigning them the class of their most similar, connected, and annotated neighbor. This analysis reduced the proportion of unclassified clustered nodes from an initial 51% to 23% (Supporting Information, Figure S14A). However, the overall class composition within a sample remained consistent. For example, in the prairie pothole sample (T3), the initial and final relative abundances of class distribution did not change significantly compared to each other, as indicated by a Bray–Curtis dissimilarity of 0.133, suggesting that the overall shift in composition was minimal due to the expanded list of annotated molecules (Supporting Information, Figure S14B). While previous studies have employed other forms of networking analysis (for instance, using sparse neighborhood and inverse covariance selection model) on py-GC/MS data without utilizing MS spectral information, we propose that incorporating MS spectra into networking analysis provides a more detailed understanding of compound relationships, especially in complex, nontargeted environmental samples.^14,74^

The distribution of individual compounds across ecosystems can also be examined through this analysis. The largest cluster in the network contains several compound classes, many of which are present in higher abundance (Figure 4D). For example, toluene and *p*-xylene, both MAHs, were found relatively in high abundance across all ecosystems with similar distribution patterns. In total, 36 nodes in the network showed contributions from all five ecosystems, while 77 nodes showed contributions from four ecosystems excluding tundra soil, representing 40.5% of the overall nodes (Supporting Information, Figure S15). However, other compounds exhibit ecosystem-specific trends and may be abundant only in certain environments. For instance, dibenzofuran and fluorene are both PAHs, but dibenzofuran was more abundant in temperate forests, while fluorene showed higher abundance in tundra samples. Some compounds were predominantly found in specific ecosystems. For example, 2,3-dimethyl-pyridine and 1-phenyl-1H-pyrrole were detected in vernal pools and prairie potholes, both wetland ecosystems. In total, we found 24 compounds from different clusters which were only present in wetlands. Pearson correlation analysis further showed that compounds from these two sites were highly correlated (*r* = 0.73, *p* < 0.001) (Figure 4E).

In the grassland soils, we also observed molecular-level differences among bulk, HF, and LF samples (Supporting Information, Figure S16A). Out of 192 total nodes, 81 contained contributions from bulk and two density fractions; however, most samples (<40% LF) aligned along the bulk–HF axis, while only a minority were strongly LF-dominated based on ternary compositional analysis (Supporting Information, Figure S16B,C). An additional 40 compounds were found exclusively in the grassland LF, clustering into two major groups, one of which was dominated by lignin-derived compounds.

## 4 Environmental Implications

Our analyses demonstrate that py-GC/MS is a powerful tool for investigating the specific composition of bulk SOM across ecosystems, in depth profiles, and in fractionation schemes (e.g., HF/LF). We streamlined py-GC/MS data analysis with an openly accessible workflow that can be easily applied to various environmental samples such as plant litter, sediments, and petroleum source rocks.^75^ Our custom compound library (available in Supporting Information) is expandable and applicable to a wide range of environmental samples, and can be modified as needed, making the workflow broadly useable. Our multistep data analysis approach, which includes spectral library matching and MS spectra-based networking analysis, allows for confident identification of compounds of interest. The parent compounds of identified pyrolysates are largely inaccessible by high-resolution techniques such as GC/HRMS or LC/HRMS but can be further validated using known standards in various HRMS. EGA analysis can be useful as a screening tool to determine thermal liability and contributions of mineral bound OC and IC pools. Importantly, data analysis was developed with high throughput in mind and virtually no sample preparation is required. This ensures that the process is very fast compared to many other SOM characterization techniques, effectively captures the composition of the entire SOM pool, and opens the door to studies examining spatial, temporal, and depth-dependent SOM composition and its impact on ecosystem function. Although large sets of samples across ecosystems are needed to fully resolve patterns and relationships between ecosystem characteristics and SOM composition, our workflow and preliminary findings highlight the promise of this approach for uncovering these links. Additional analyses, such as ^14^C analysis of organic compounds, can help disentangle the factors driving SOM cycling rates and persistence.

We showed that some SOM compound class distributions can be attributed to microbial and plant origins, providing a framework that can be further extended by developing a more comprehensive molecular data set containing compounds with confirmed origins. Additionally, we observed depth-dependent changes in SOM composition. Furthermore, distinctions among density fractions are critical for understanding SOM stability. Our study demonstrates that py-GC/MS remains a vital tool for the characterization of SOM, and that improved data analysis pipelines can extend the number of identified compounds used to interpret SOM reactivity and stability. For example, studies have shown that the chemical energy in SOM can be altered due to differences in land use, organic inputs, and microbial community composition.^76–78^ However, these studies do not take the total pool of SOM into account when making predictions of C stability and turnover, often relying on extractions of a small portion of total SOM (30–40%). In addition, py-GC/MS has been used to investigate the influence of SOM on contaminant transport and behavior in soils.^79–82^ Understanding the composition and cycling of SOM is vital for predicting the behavior of trace elements, heavy metals, and organic contaminants. Our pipeline could be helpful to provide valuable insights into how SOM composition affects metal binding and the partitioning of organic contaminants in soils.

Through this work, we have opened several avenues for future research into the composition, diversity, origin, and stability of SOM across ecosystems. This high-throughput approach can be used to begin building a much more detailed picture of SOM composition across space and time and, in turn, help improve models of regional and global biogeochemical C cycles.

## Supplementary Material





## Notes

Compound classes library data (XLSX)
Additional information on sample site and collection, density fractionation, analytical method, statistical analysis, method development results, organic standards result, class distribution and clustering analysis of additional samples, EGA/MS of additional samples and standards, EGA/MS results of acid treated samples, networking analysis of density fractionated samples (PDF)
