# Supplementary material for: Optimizing Cryo-Focused Pyrolysis GC/MS for Tracing Soil Organic Matter Across Diverse Ecosystems
Source: Environ Sci Technol. 2026 Mar 16;60(12):9237–49. doi: 10.1021/acs.est.5c16415 (PMC13045018; doi:10.1021/acs.est.5c16415)
Supplement: Supplementary file 2 [file es5c16415_si_002.pdf]

Supporting Information

Optimizing Cryo-focused Pyrolysis GC/MS for Tracing Soil Organic Matter Across Diverse Ecosystems

Abrar Shahriar[1*], Mavrik Zavarin[1], Erin E. Nuccio[1], Katherine E. Grant[1], Karis J. McFarlane[1], Jennifer Pett-Ridge[1,2,3], Daniel Toews[2], Jason P. Sexton[2], Rebecca Ryals[2], Joy Baccei[4], Aiden (AJ) Berndt[5], Rene Boiteau[5], Ishtiaq Ahmed Jawad[6], Yu Yang[6], Sheel Bansal[7], Edward J. O'Loughlin[8], Kenneth M. Kemner[8], Roser Matamala[9], Keith D. Morrison[1*]

[1]Physical and Life Sciences Directorate, Lawrence Livermore National Laboratory, Livermore, CA 94550, USA

[2]Department of Life and Environmental Sciences, University of California, Merced, Merced, CA 95343, USA

[3]Innovative Genomics Institute, University of California, Berkeley, Berkeley, CA 94720, USA

[4]University of California Natural Reserve System, Merced, CA 95343, USA

[5]College of Science and Engineering, University of Minnesota, Minneapolis, MN 55455, USA

[6]Department of Civil and Environmental Engineering, University of Nevada, Reno, Reno, NV 89557, USA

[7]U.S. Geological Survey, Northern Prairie Wildlife Research Center, Jamestown, ND 58401, USA

[8]Biosciences Division, Argonne National Laboratory, Lemont, IL 60439, USA

[9]Environmental Science Division, Argonne National Laboratory, Lemont, IL 60439, USA

*Corresponding authors

Email: shahriar1@llnl.gov

Email: morrison30@llnl.gov

Number of pages: 25

Number of tables: 2

Number of figures: 16

Content

Text S1. Soil Samples Collection……………………………………………………………………...................... *S3*
Text S2. Density Fractionation of Soil Samples……………………………………………………………….. *S3*
Text S3. Organic Standards……………………………………………………………………………………. *S4*
Text S4. Single shot py-GC/MS analysis using DB-Wax column…………………………………………….. *S4*
Text S5. Compound class assignment criteria………………………………………………………………. *S5*
Text S6. Expansion of annotations in networking analysis…………………………………………………. *S5*
Text S7. Molecular diversity indices calculation…………………………………………………………… *S5*
References……………………………………………………………………………………………….. *S6*
Table S1. Site information for all soil samples.……………………………………………………………….. *S7*
Table S2. Compound classification details…………………………………………………………………. *S9*
Figure S1. TIC of vernal pool samples using DB-Wax and HP-5MS column, respectively………………….. *S10*
Figure S2. TIC of **A)** vernal pool (upland, 10-30 cm depth) samples having different amounts of total C. **B)** vernal pool (center 0-10 cm depth) sample with very high C concentration…………………………………... *S11*
Figure S3. Compound class distribution of lignin, humic acid, L-tryptophan standard……………………......... *S12*
Figure S4. **A**) Distribution of the number of compounds identified in each class. **B)** Hierarchical clustering of the samples ……... *S13*
Figure S5. A) Class distribution of different prairie pothole samples. **B)** Hierarchical clustering of the six samples collected from prairie potholes……………………………………………………………………….. *S14*
Figure S6. Class distribution of three different tundra soil samples………………………………………….. *S15*
Figure S7. A) Class distribution of different depth and location vernal pool samples. **B)** Hierarchical clustering of the six samples (three sites, two depth) collected from vernal pools. **C)** Shannon diversity index and richness of the SOM for six samples……………………….. *S16*
Figure S8. EGA/MS curve and MS spectra at the highest temperature of temperate forest (0-5 cm depth sample) and vernal pool (upland, 0-10 cm depth sample), respectively……………………………………….. *S17*
Figure S9. A) EGA of tundra soil (SC1 sample). **B)** MS spectra at the highest peak of EGA curve (at 458.3 °C). **C)** MS spectra at 650 °C………………………………………………………………………………. *S18*
Figure S10. EGA/MS curve of humic acid, $CaCO_3$, and humic acid+ $CaCO_3$ mixture..……………………… *S19*
Figure S11. A) EGA chromatogram of prairie pothole samples (P1) before and after acid treatment by 3N HCl. **B)** Comparison between no treatment bulk grassland soil and acid insoluble grassland soil sample's EGA/MS curve………………………………………………….. *S20*
Figure S12. XIC of CO_2 in bulk, HF, and LF fraction in grassland soil samples (0-10 cm depth)…………… *S21*
Figure S13. A) Histogram showing the distribution of pairwise cosine similarity values and the corresponding number of nodes. **B)** Bar chart showing the total number of edges at different pairwise cosine similarity thresholds……………………………………………………………………………………. *S22*
Figure S14. A) Changes of number of annotated nodes in overall networking analysis based on python workflow. **B)** Class distribution of prairie pothole sample before and after of expanded annotation................ *S23*
Figure S15. Quantitative representation of compounds across different ecosystems. ………………………... *S24*
Figure S16. A) Molecular networking analysis of three different density fractions (bulk, HF, and LF) of grassland soil samples. **B)** quantitative representation of distribution of compounds in different density fractions. **C)** 2D ternary projection of Bulk, HF, and LF compositions…………………………………….. *S25*

Text S1. Soil Sample Collection

Vernal pool samples were collected in August 2024 from a vernal pool site (details in **Table S1**) located at the University of California, Merced. Samples were taken from three distinct landscape positions (upland, edge, and center; 15 m apart) and two depths (0–10 cm and 10–30 cm) using freshly dug soil pits in an ephemeral vernal pool. Samples were transported on dry ice in sealed plastic bags, then air-dried, homogenized, passed through a 2 mm sieve, and stored at room temperature.

Prairie pothole samples were collected in August 2024 from the Cottonwood Lake Study Area (CLSA), located northwest of Jamestown, North Dakota. This protected site, managed by the U.S. Fish and Wildlife Service, has served as a long-term research area (>30 years) for the U.S. Geological Survey (USGS). Detailed site descriptions and maps are available in the corresponding USGS professional paper.[1] Samples were collected from upland zones of both permanent (P sites) and temporary (T sites) wetlands, with only 0–10 cm depth samples analyzed. Samples were transported on dry ice in plastic bags, then air-dried, homogenized, and stored at room temperature following the same procedure as the vernal pool samples.

Temperate forest soil samples were collected from Harvard Forest, Massachusetts. Only bulk soil samples from the 0–5 cm depth were used for this study. Site details and collection procedures are described in McFarlane et al.[2] Tropical Forest soils were collected from the Luquillo Experimental Forest in Puerto Rico. Although Mayer et al.[3] collected and analyzed soil samples from multiple locations and depths in their study, only 0–10 cm upland valley samples were analyzed for this study. Additional site and sample collection details can be found in Mayer et al.[3]

Tundra soil samples were collected from Unalaska Island in Alaska. Total three samples were analyzed, and all those samples were 0–10 cm depth samples. The samples were first freeze-dried, then homogenized and sieved prior to analysis. Wildfire affected boreal forest soil sample was collected from Red Lake, Ontario, Canada in August 2023.

Soil samples were collected from the University of California's Hopland Research and Extension Center (HREC) in January 2022. The site consists of a lightly grazed annual grassland with a Mediterranean-type climate. For this study, samples from four depth intervals (0–10, 10–20, 20–50, and 50–100 cm) were analyzed. Further details regarding the site and sampling procedures are provided in Grant et al.[4]

Text S2. Density Fractionation of Soil Samples

Grassland soil samples from different depths were density fractionated using a low-carbon and low-nitrogen sodium polytungstate solution (SPT-O, Geoliquids) into three fractions: the free light fraction (FLF), the occluded light fraction (OLF), and the heavy fraction (HF). However, for the purposes of this study, only the FLF was used to represent the light fraction (LF). A detailed description of the density fractionation procedure is provided in Grant et al.[4]

Briefly, a 1.65 g/mL SPT solution was used. Twenty grams of 2 mm-sieved soil were transferred into a centrifuge tube containing 100 mL of the SPT solution. Samples were centrifuged at 3500 rpm for 1 hour, after which the floating material (FLF) was aspirated and collected by vacuum filtration. The washed FLF was dried overnight in a 65 °C oven, weighed, then transferred to a 105 °C oven for further drying, and subsequently ground.

After recovering the OLF from the centrifuge tubes as described in Grant et al.[4], the remaining material was designated as HF. The HF was rinsed repeatedly until the supernatant reached a density of 1 g/mL. Samples were then dried overnight at 65 °C, weighed, further dried at 105 °C, ground using a ball mill, and transferred to vials for later analysis. Density fractionation was performed in triplicate.

Text S3. Organic Standards

Different organic standards were used for method development purpose in this study. Kraft lignin (Alkali, 370959) was purchased from Sigma-Aldrich (Bellefonte, PA, USA). Humic acid (Suwannee River Humic Acid Standard III, 3S101H) was purchased from International Humic Substances Society (IHSS, St. Paul, MN, USA). L-Tryptophan (99.0-101.0%) was also purchased from Sigma-Aldrich.

Text S4. Single shot py-GC/MS analysis using DB-Wax column

Same py-GC/MS system was used as described in the main text for running sample with Agilent DB-Wax-UI column (30m × 250μm inside diameter × 0.25μm inner coating) column. The AGC oven was held at 40 °C for 5 min and then heated at 2 °C/min to 200 °C then held for 5 min. After the first heating ramp, the oven was heated rapidly at 20 °C/min to 250 °C. The inlet to the GC-MS was kept at 300 °C and the septum purge flow was 3 mL/min. The inlet was run in split mode, with the gases entering the EGA column at a split flow of 20:1 (He: sample gas). The mass spectrometer was set to scan on the first quadrupole mass spectrometer from *m/z* 29 to 850, using a 300 ms scan time.

Text S5. Compound class assignment criteria

The library was constructed from more than 1,600 compounds detected across all samples analyzed in this study. Compounds were classified based on their chemical structures, as described in Table S2, which also provides implications regarding their potential origins. Common contaminants (including, but not limited to, siloxanes, phthalates, sulfonates) were excluded from the library. Halogenated compounds and other potentially synthetic compounds that are unlikely to represent SOM were also omitted. The complete list of compounds is provided as a compound library Excel file.

Text S6. Expansion of annotations in networking analysis

A Python script was developed to expand the annotated feature list of MS data by mining results generated through GNPS networking. The script operates directly on the node and edge tables produced in Cytoscape during the networking analysis. First, it processes all nodes to assign an initial chemical classification based on the original NIST annotations. Next, the script identifies nodes lacking an initial classification and examines the edge data to determine which classified nodes are connected to each 'Unknow' node. For each 'Unknown' node, it compiles all edges linked to nodes with known classifications and assigns the classification associated with the highest cosine score, based on the MS spectra of each feature. If all connected nodes are classified as 'Unknown' the script outputs 'Unknown' as the final classification.

Text S7. Molecular diversity indices calculation

The Shannon diversity index was calculated based on equation 1 where Py refers to the relative abundance of each pyrolysate compound based on their integrated peak heights.[5,6] The richness is defined as the total number of unique compounds found in each sample.

$$\text{Shannon index, H} = \sum_{i=1}^{S} (Py_i \times \ln Py_i) \quad \text{.................................(1)}$$

The Simpson diversity index was calculated based on equation 2 where n represents the number of unique pyrolysate compounds under the same class whereas N represents the total number of classed compounds.[7]

$$\text{Simpson's index, D} = \frac{\sum_{i=1}^{S} n_i(n_i - 1)}{N(N-1)} \quad \text{.................................(2)}$$

Code and Data Availability

All the codes and data associated with this study can be made available upon request.

References

(1) Mushet, D. M.; Jr, N. H. E.; Rosenberry, D. O.; LaBaugh, J. W.; Bansal, S.; Levy, Z. F.; McKenna, O. P.; McLean, K.; Mills, C. T.; Neff, B. P.; Nelson, R. D.; Solensky, M. J.; Tangen, B. *Lessons Learned from Wetlands Research at the Cottonwood Lake Study Area, Stutsman County, North Dakota, 1967–2021*; 1874; U.S. Geological Survey, 2022. https://doi.org/10.3133/pp1874.

(2) McFarlane, K. J.; Torn, M. S.; Hanson, P. J.; Porras, R. C.; Swanston, C. W.; Callaham, M. A.; Guilderson, T. P. Comparison of Soil Organic Matter Dynamics at Five Temperate Deciduous Forests with Physical Fractionation and Radiocarbon Measurements. *Biogeochemistry* **2013**, *112* (1), 457–476. https://doi.org/10.1007/s10533-012-9740-1.

(3) Mayer, A. C.; McFarlane, K. J.; Silver, W. L. The Effect of Repeated Hurricanes on the Age of Organic Carbon in Humid Tropical Forest Soil. *Glob. Change Biol.* **2024**, *30* (4), e17265. https://doi.org/10.1111/gcb.17265.

(4) Grant, K. E.; Repasch, M. N.; Finstad, K. M.; Kerr, J. D.; Marple, M.; Larson, C. J.; Broek, T. A. B.; Pett-Ridge, J.; McFarlane, K. J. Diverse Organic Carbon Dynamics Captured by Radiocarbon Analysis of Distinct Compound Classes in a Grassland Soil. *Biogeosciences* **2024**, *21* (19), 4395–4411. https://doi.org/10.5194/bg-21-4395-2024.

(5) Song, F.; Hu, N.; Lou, Y.; Zhang, H.; Zhu, P.; Li, D.; Gao, H.; Zhang, S.; Wang, Y. Divergent Chemical Compositions of Soil Organic Matter Size Fractions under Long-Term Amendments across a Climate Gradient. *Soil Tillage Res.* **2024**, *242*, 106156. https://doi.org/10.1016/j.still.2024.106156.

(6) Wang, Y.; Wang, S.; Liu, C.; Zhu, E.; Jia, J.; Feng, X. Shifting Relationships between SOC and Molecular Diversity in Soils of Varied Carbon Concentrations: Evidence from Drained Wetlands. *Geoderma* **2023**, *433*, 116459. https://doi.org/10.1016/j.geoderma.2023.116459.

(7) Jones, A. R.; Dalal, R. C.; Gupta, V. V. S. R.; Schmidt, S.; Allen, D. E.; Jacobsen, G. E.; Bird, M.; Grandy, A. S.; Sanderman, J. Molecular Complexity and Diversity of Persistent Soil Organic Matter. *Soil Biol. Biochem.* **2023**, *184*, 109061. https://doi.org/10.1016/j.soilbio.2023.109061.

Any use of trade, firm, or product names is for descriptive purposes only and does not imply endorsement by the U.S. Government.

Table S1. Site information for all soil samples.

Ecosystem	Location	Site/Sample Description	Site name	Coordinates (Latitude, Longitude)	Depth (cm)	Total C%	Total N%	C:N
Vernal Pools	Merced, CA, USA	Upland		37°22'11.5"N 120°24'40.2"W	0–10	4.42	0.32	13.68
					10–30	6.05	0.52	11.56
		Transition			0–10	6.21	0.56	11.10
					10–30	1.00	0.11	9.09
		Center			0–10	1.23	0.13	9.49
					10–30	0.89	0.09	9.85
Prairie Potholes	Cottonwood Lake, Jamestown, ND, USA	Permanent	P1	47°06'01.0"N 99°05'55.0"W	0–10	3.16	0.11	29.59
			P6	47°05'48.4"N 99°05'44.0"W	0–10	1.77	0.07	26.60
			P8	47°06'01.8"N 99°06'18.6"W	0–10	5.43	0.32	16.97
		Temporary	T3	47°05'59.0"N 99°05'49.0"W	0–10	9.94	0.72	13.75
			T6	47°05'55.6"N 99°05'38.0"W	0–10	3.90	0.38	10.35
			T9	47°05'47.8"N 99°05'31.8"W	0–10	2.62	0.28	9.46
Temperate Forest	Harvard Forest, MA, USA			42°32'15.7"N 72°10'17.4"W	0–5	10.00	0.38	26.32
Tropical Forest	Luquillo Experimental Forest, PR, USA			18°18'56.5"N 65°44'55.3"W	0–10	5.20	0.55	14.18

Tundra	Unalaska, AK, USA		SC1	53°54'30.4"N 166°25'53.6"W	0–10	6.53	0.52	12.55
			SC3	53°50'17.7"N 166°33'16.3"W	0–10	10.16	0.55	18.64
			SC8	53°50'48.2"N 166°27'42.9"W	0–10	3.15	0.19	16.55
Wildfire-affected boreal forest	Red Lake, Ontario, Canada	Burned		51°18'42.5"N 93°22'21.5"W	0–15	34.51	1.13	30.68
Grassland	Hopland, CA, USA	Bulk soil		39°00'03.6"N 123°04'08.4"W	0–10	3.14	0.27	11.70
					10–20	1.22	0.13	9.46
					20–50	0.50	0.09	5.88
					50–100	0.25	0.07	3.79
		Heavy density fraction			0–10	1.78	0.18	9.69
					10–20	1.03	0.12	8.44
					20–50	0.48	0.08	5.75
					50–100	0.23	0.06	3.75
		Light density fraction			0–10	28.53	1.08	26.44
					10–20	28.05	0.81	34.78
					20–50	28.92	0.89	32.39

Table S2. Compound classification details.

Chemical Class	Description	Example compounds	Number of compounds in library
Alkenes	Hydrocarbon containing a C–C double bond	1-Nonene, 1-Octene, 1-Octene, 3,7-dimethyl-	141
Degraded saccharides	Compounds derived from partially degraded saccharides	Furan,2-methyl-, Furan,3-methyl-, Cyclopentane,methylene-	77
Fatty acids, alcohols, and esters,	Hydrocarbon chain containing −COOH, −OH, or R−C(=O)−O−R' functional group. Also includes carbonyl compounds from fatty acids	Palmitoleicacid, Myristicacidglycidylester, Octadecanoic acid, 2-methylpropyl ester	159
Lignin	Alkoxy benzene and phenolic compounds	Phenol,2-methoxy, Acetophenone, 3-Ethenylphenol	74
Long alkanes	Hydrocarbon containing a C–C single bond where C ≥ 22	Tetracosane, Pentacosane, Hexacosane	18
Monocyclic aromatic hydrocarbons (MAH)	Aromatic hydrocarbon containing single benzene ring. No N is present in the compound.	Benzene, Toluene, Ethylbenzene	265
N-containing monocyclic aromatic hydrocarbons (N-MAH)	MAH with N	Benzonitrile, Benzene,isocyanato-, Benzonitrile,2-methyl-	172
Other hydrocarbons	Various cyclo-hydrocarbon	1,3-Cyclopentadiene, 1,4-Cyclohexadiene, 1,3-Cyclopentadiene,1-methyl-	124
Other N-containing compounds	N containing compounds without aromatic benzene ring.	Acetonitrile, 2-Propenenitrile, Propanenitrile	345
Phenols	Aromatic compounds containing −OH group but not alkoxy phenol	Phenol, Phenol,2-methyl-, Phenol,3-methyl-	42
Polycyclic aromatic hydrocarbons (PAH)	Aromatic hydrocarbon containing multiple benzene rings	Naphthalene, Anthracene, Pyrene	168
Short alkanes	Alkanes with C ≤ 22	Octane, Nonane, Decane	93

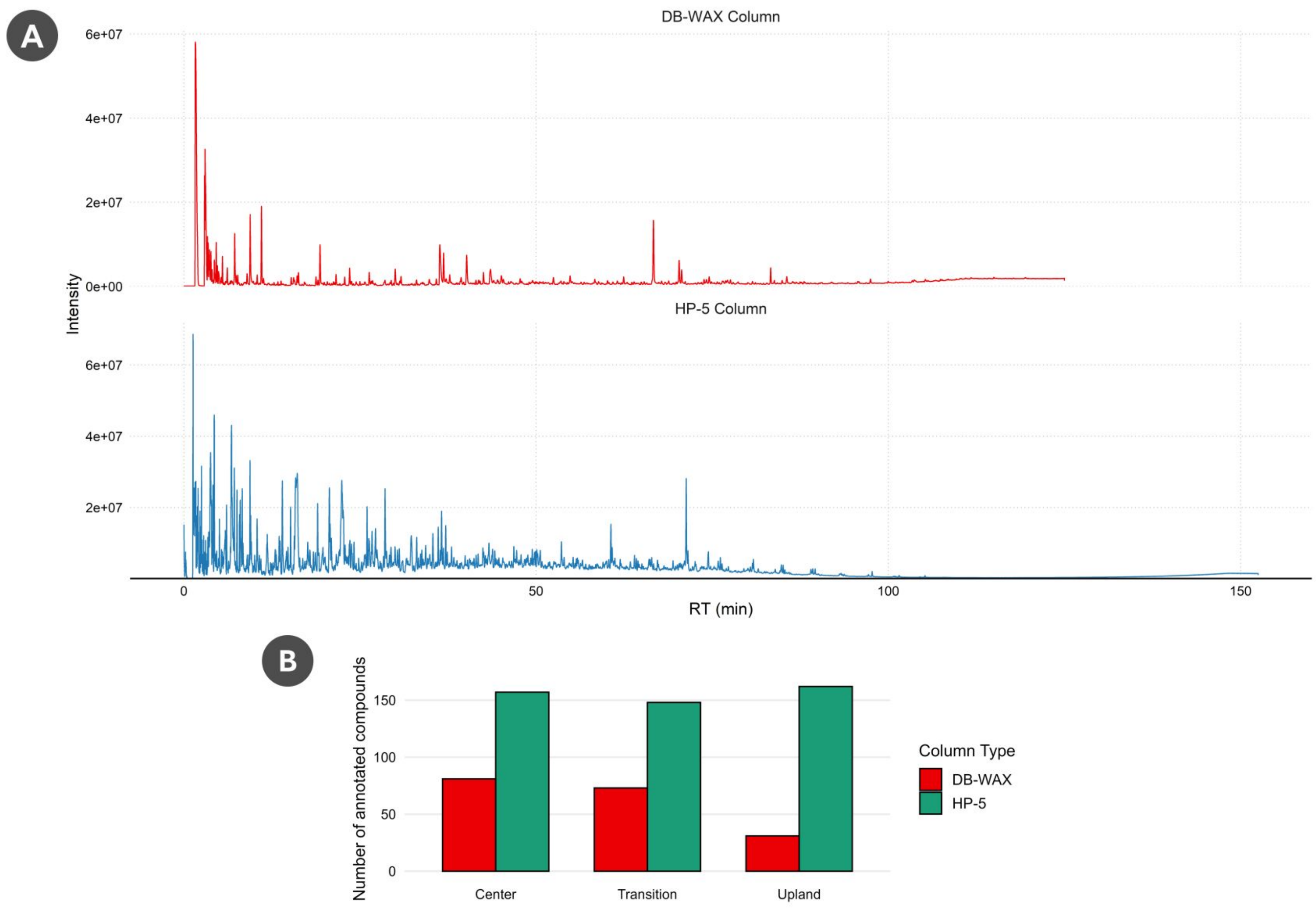

Figure S1. A) Total ion chromatogram (TIC) of vernal pool (upland, 0-10 cm depth) samples using DB-Wax and HP-5MS column, respectively. Both cases, identical weight of samples were used although the total run time was not same for the methods as described in main text and SI. **B)** Comparison between two different columns in terms of total number of annotated compounds based on three vernal pool sites (0-10 cm depth).

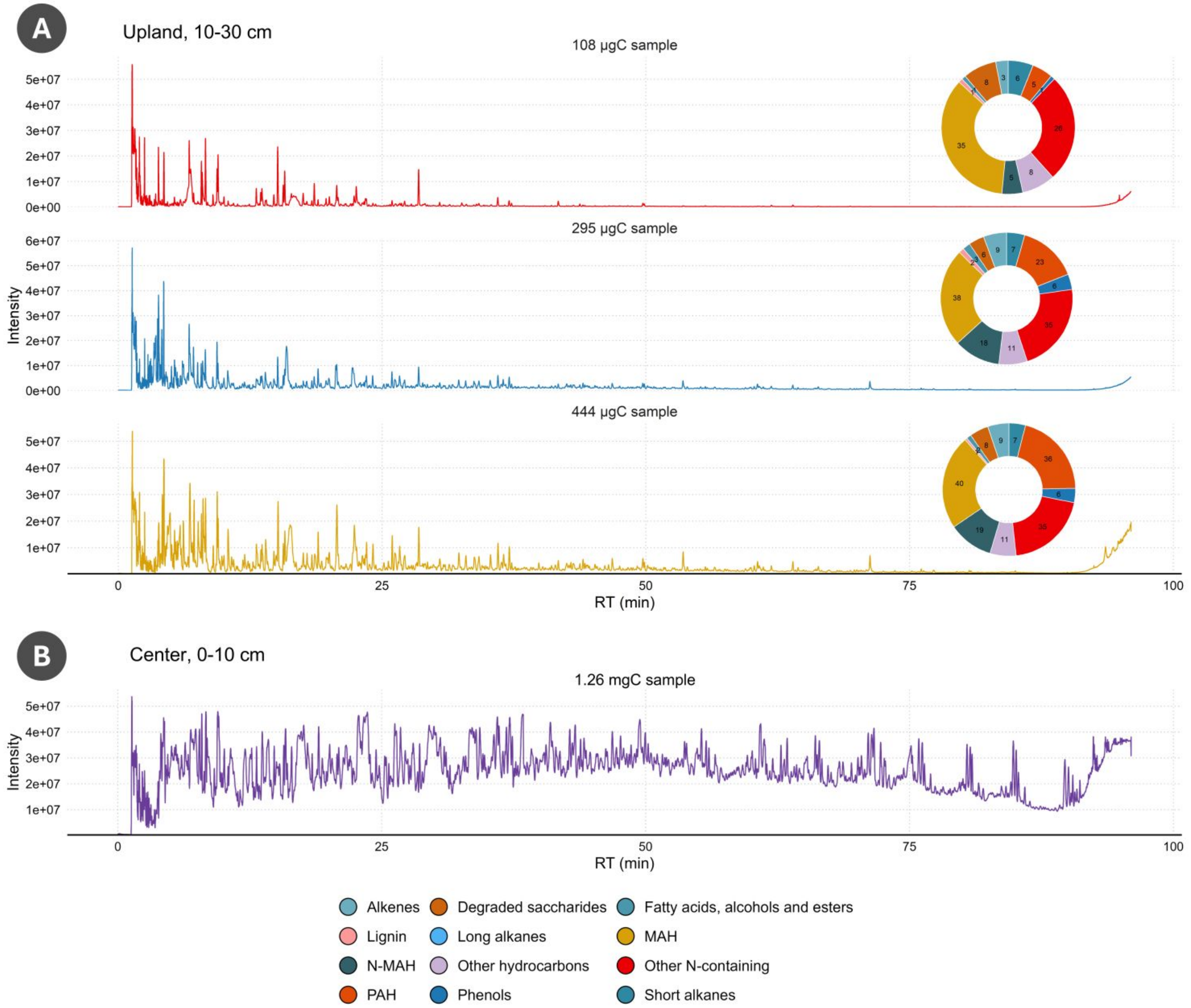

Figure S2. Total ion chromatogram (TIC) of **A)** vernal pool (upland, 10-30 cm depth) sample having different amounts of total C. The donut chart here represents the total number of unique compounds that were identified under the same compound classes in these three samples with different C concentration. **B)** vernal pool (center 0-10 cm depth) sample with very high C concentration.

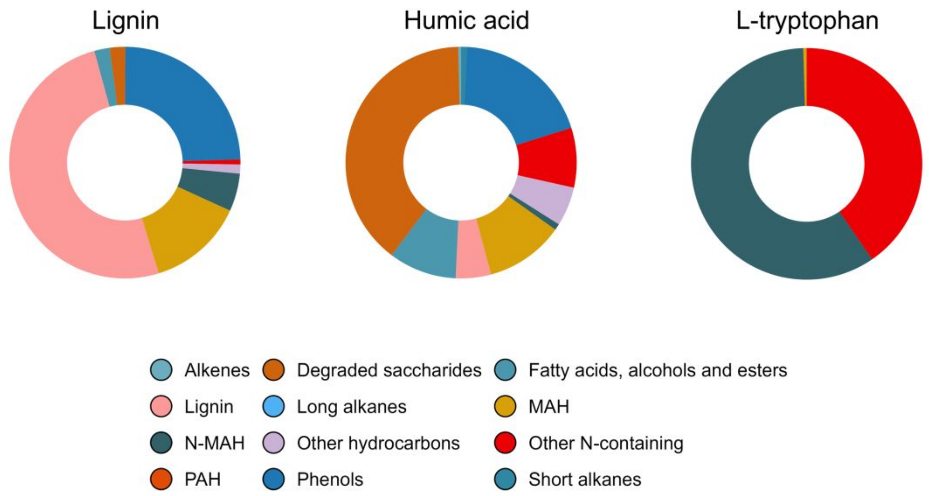

Figure S3. Compound class distribution of lignin, humic acid, and L-tryptophan standard. The donut chart represents the compound classes normalized to their total abundance.

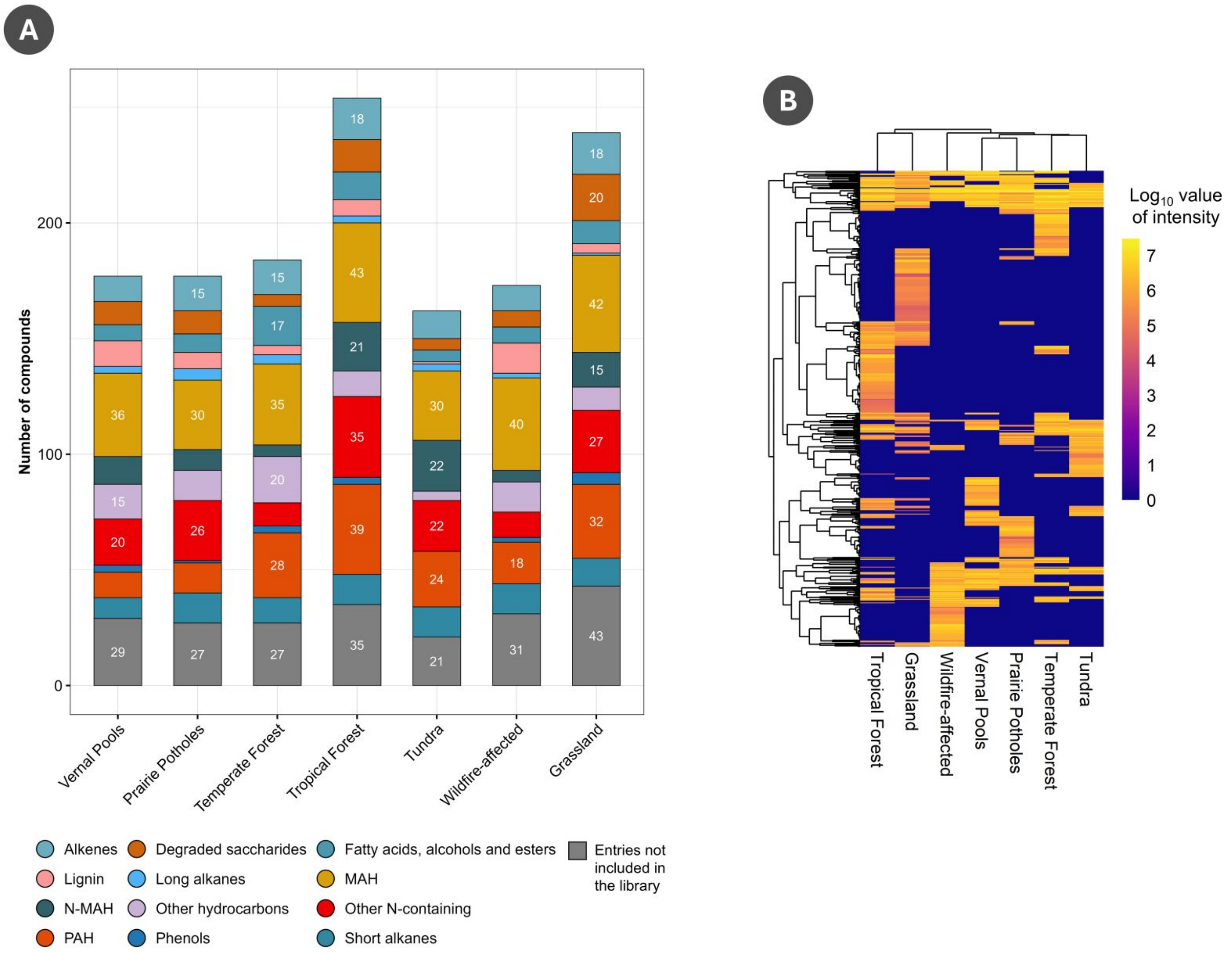

Figure S4. A) Distribution of the number of compounds identified in each class for the samples shown in Figure 2 of the manuscript. **B)** Hierarchical clustering of the samples shown in Figure 2 of the manuscript. The heatmap colors represent the log-transformed average intensity of each pyrolysate.

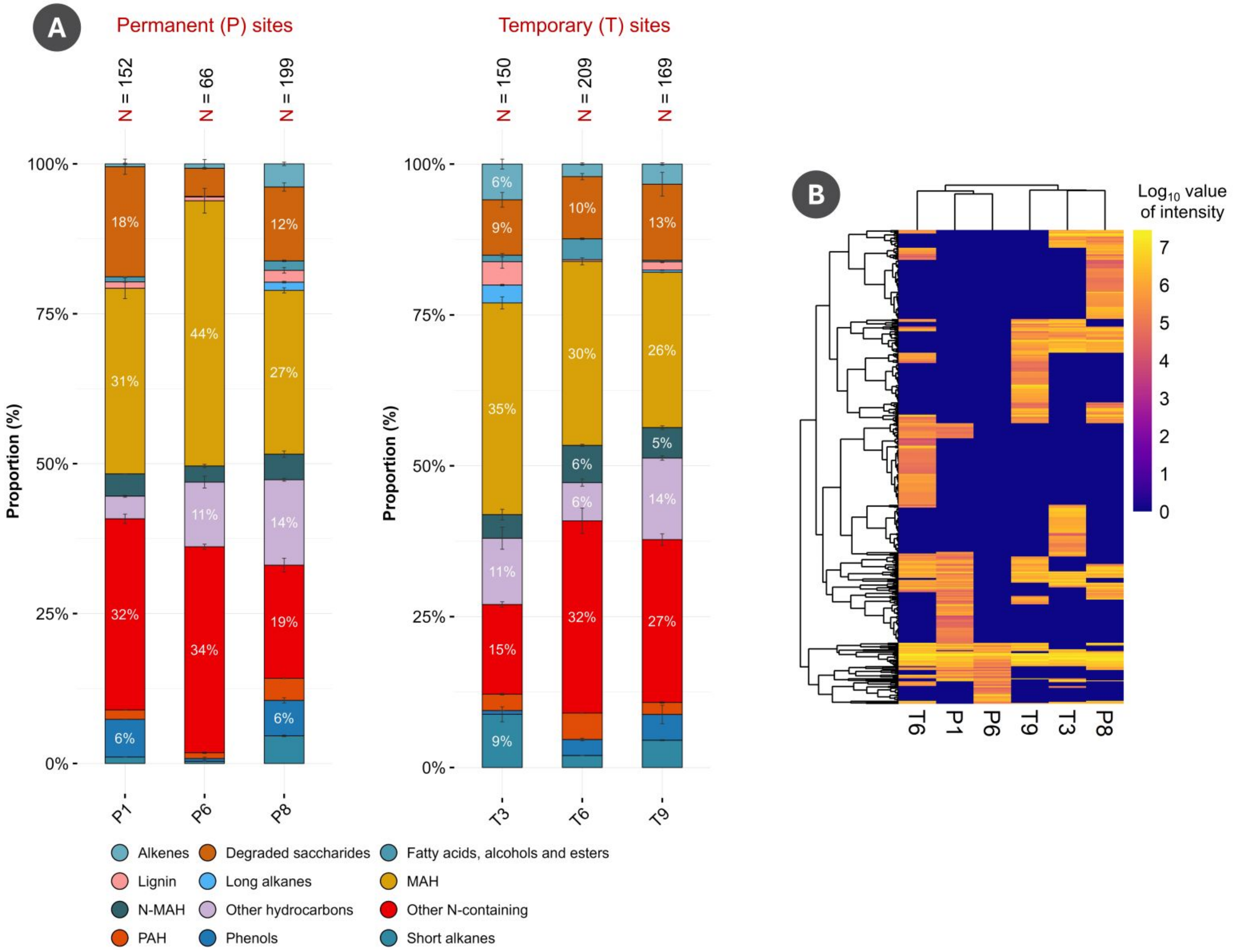

Figure S5. A) Relative abundance of compound classes in prairie pothole samples. P and T denote permanent and temporary sites, respectively. The x-axis shows three sampling locations under each site, and N indicates the total number of unique compounds identified per site. **B)** Hierarchical clustering of six prairie pothole samples. The heatmap colors represent the log-transformed average intensity of each pyrolysate**.**

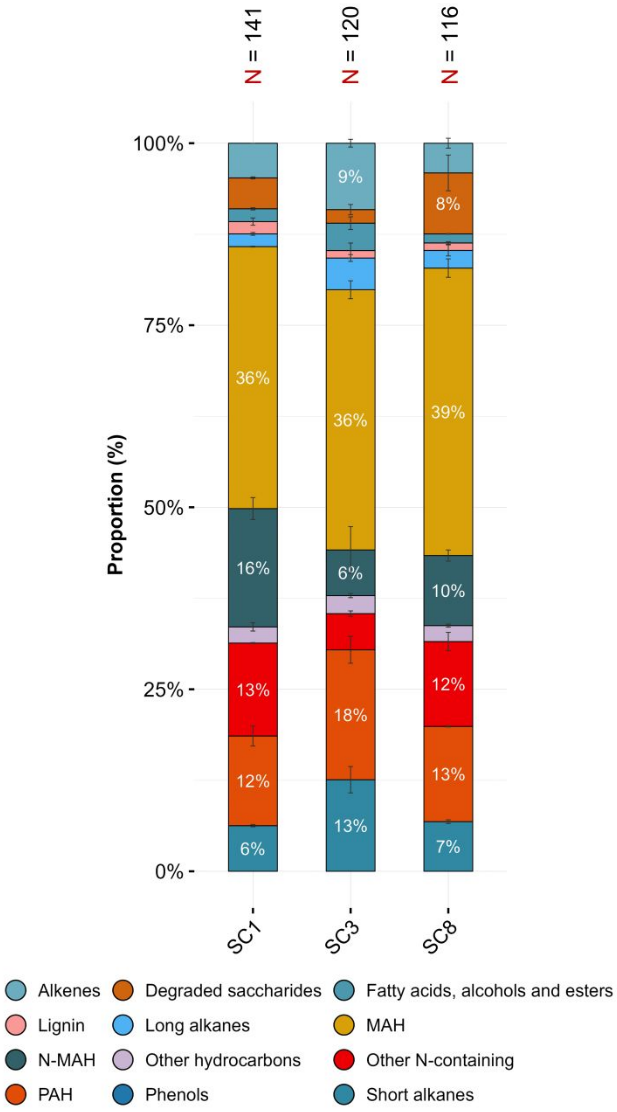

Figure S6. Relative abundance of compound classes in three different tundra soil samples. N indicates the total number of unique compounds identified per site.

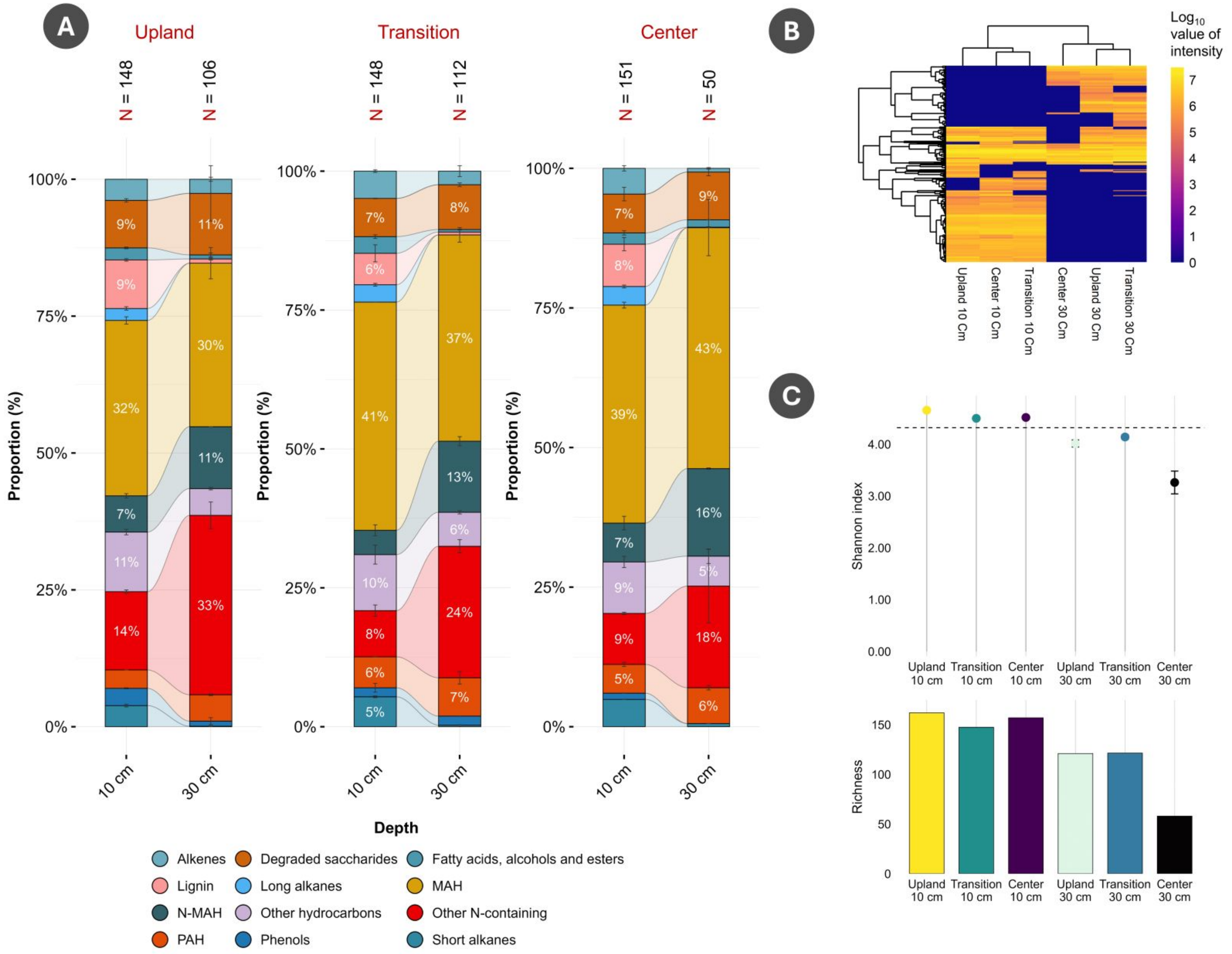

Figure S7. A) Distribution of compound classes across different depths and sampling locations in vernal pool samples. **B)** Hierarchical clustering of six vernal pool samples (three sites, two depths). The heatmap colors represent the log-transformed average intensity of each pyrolysate **C)** Shannon diversity index and richness of SOM for six vernal pool samples (three sites, two depths).

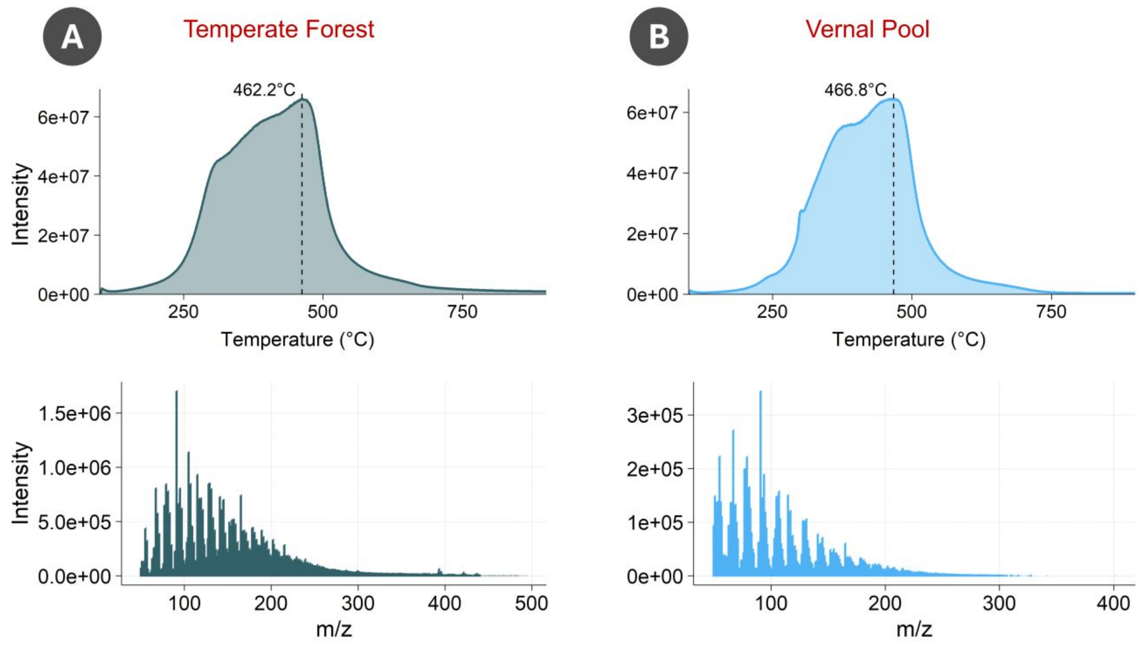

Figure S8. Evolved gas analysis (EGA) curve and mass spectra (MS) spectra at the highest temperature of **A)** temperate forest (0-5 cm depth sample) and **B)** vernal pool (upland, 0-10 cm depth sample), respectively.

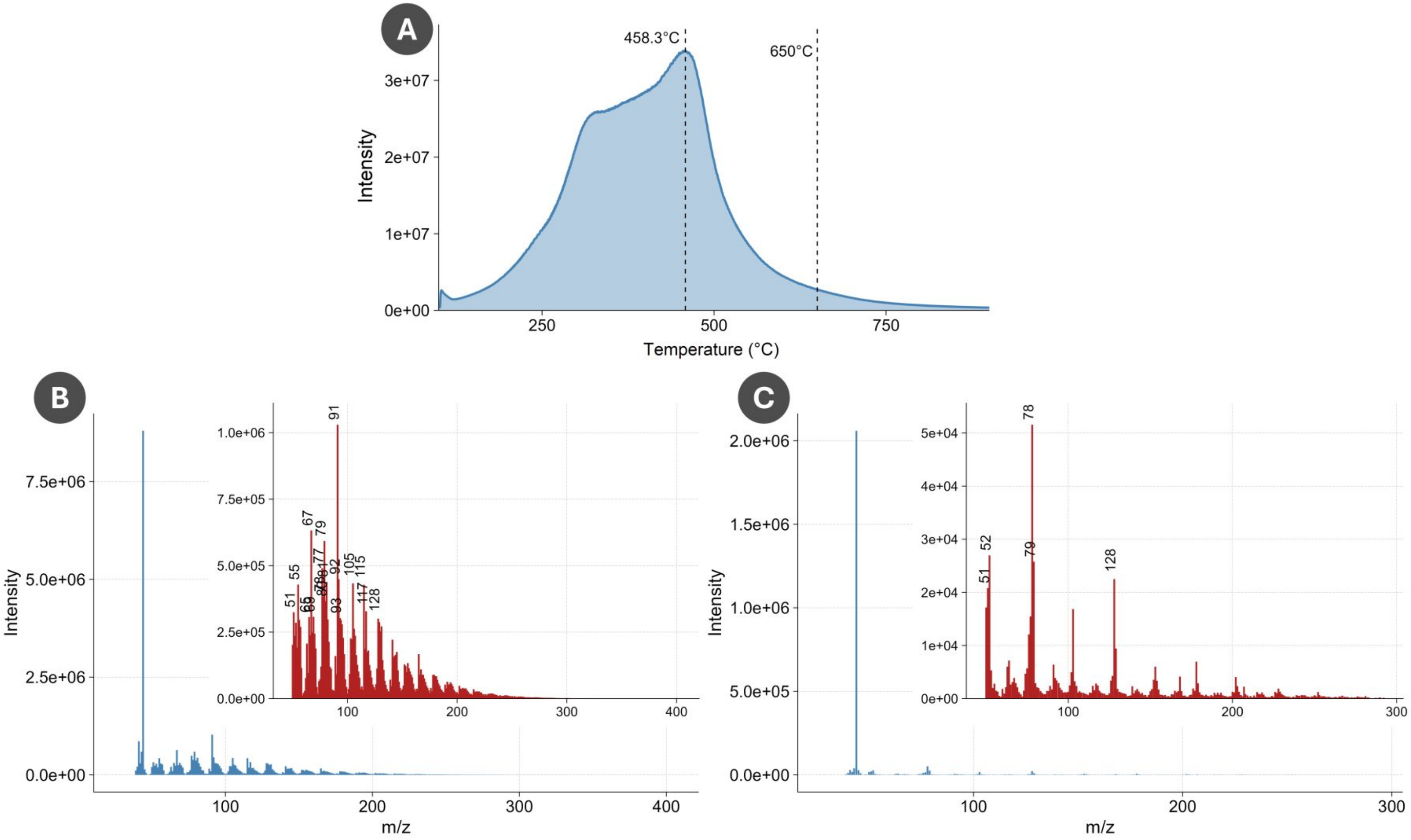

Figure S9. A) Evolved gas analysis (EGA) curve of tundra soil (SC1 sample). **B)** mass spectra (MS) spectra at the highest peak of EGA curve (at 458.3 °C). **C)** MS spectra at 650 °C. The main panels B and C display the full MS spectra, whereas the insets show the spectra starting from *m/z* = 50 to provide a clearer view of the less abundant peaks.

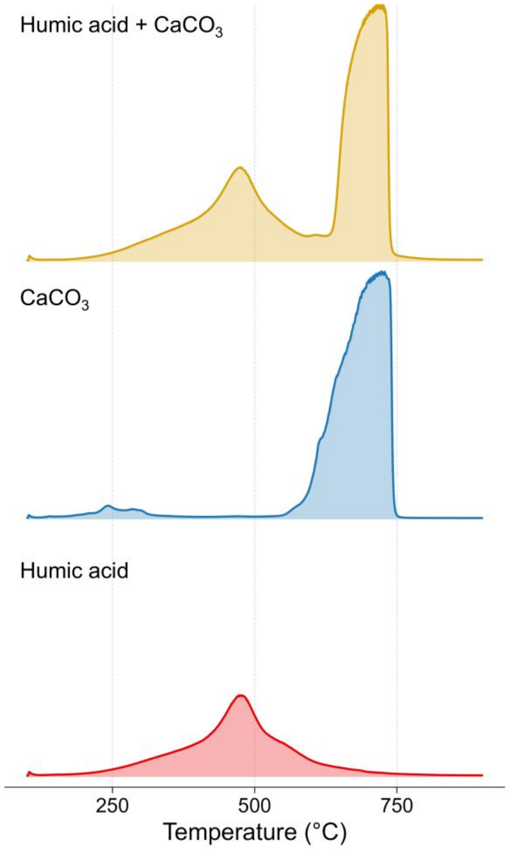

Figure S10. Evolved gas analysis (EGA) curve curve of humic acid, $CaCO_3$, and humic acid+ $CaCO_3$ mixture. The y-axis (not shown in the figure) represents the intensity in the same scale for each panel.

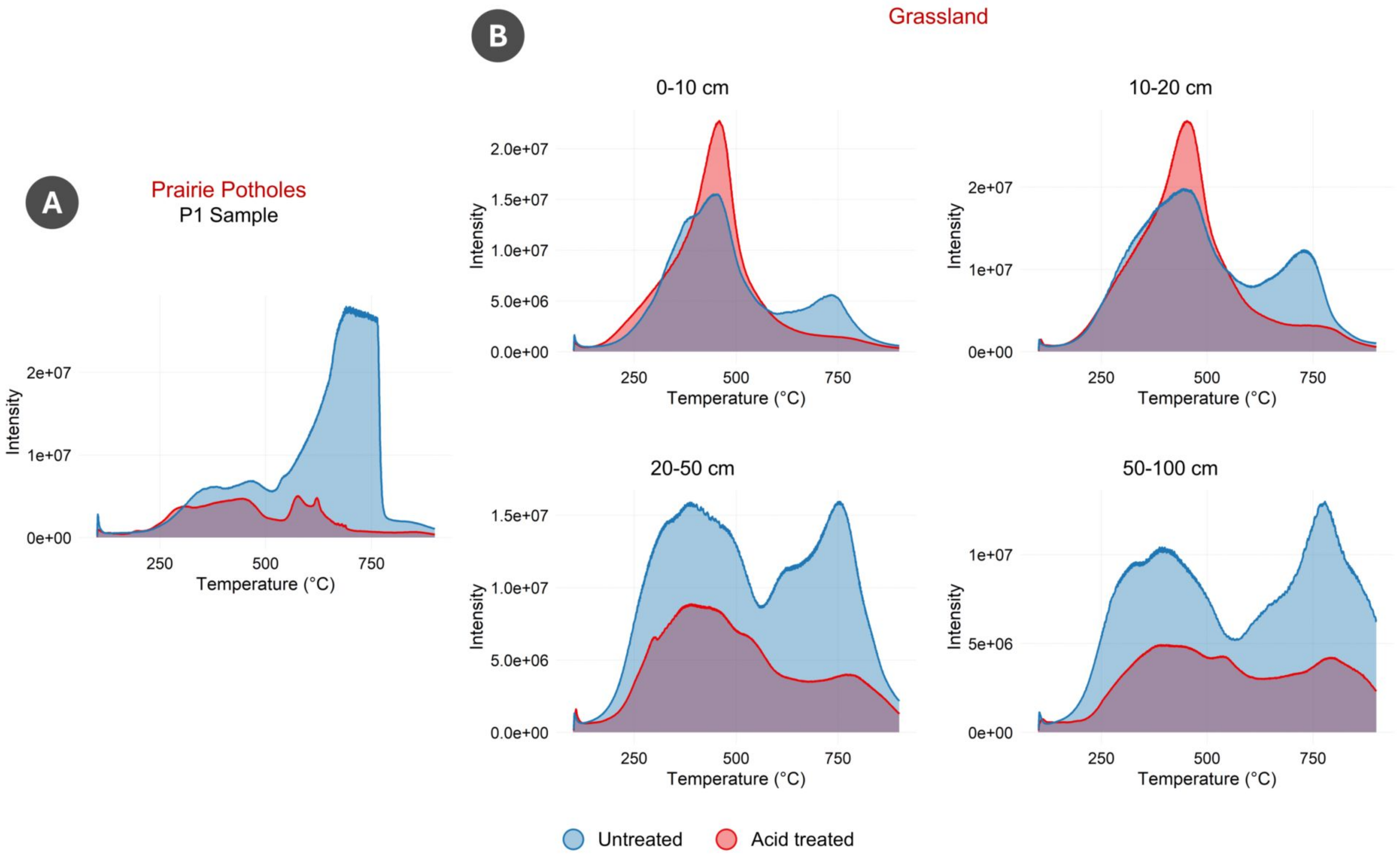

Figure S11. A) Evolved gas analysis (EGA) curve of prairie pothole samples (P1) before and after acid treatment by 3N HCl. **B)** Comparison of EGA curves between untreated bulk grassland soil and acid-insoluble grassland soil samples.

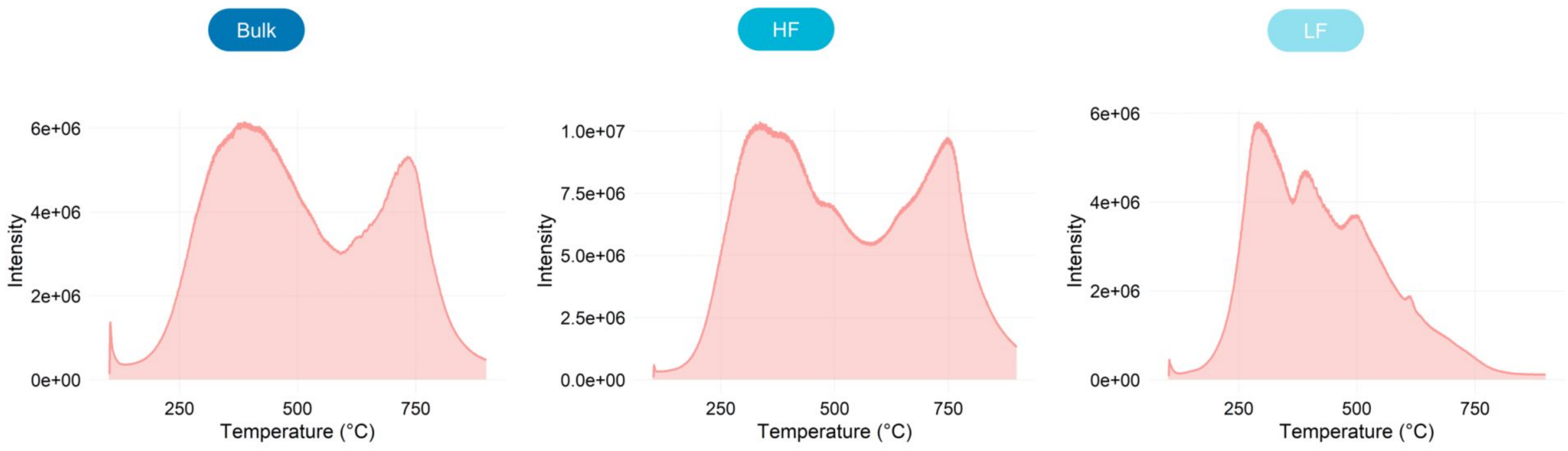

Figure S12. Extracted ion chromatogram (XIC) of CO_2 (m/z = 44) in the bulk, HF, and LF fraction of grassland soil samples (0-10 cm depth).

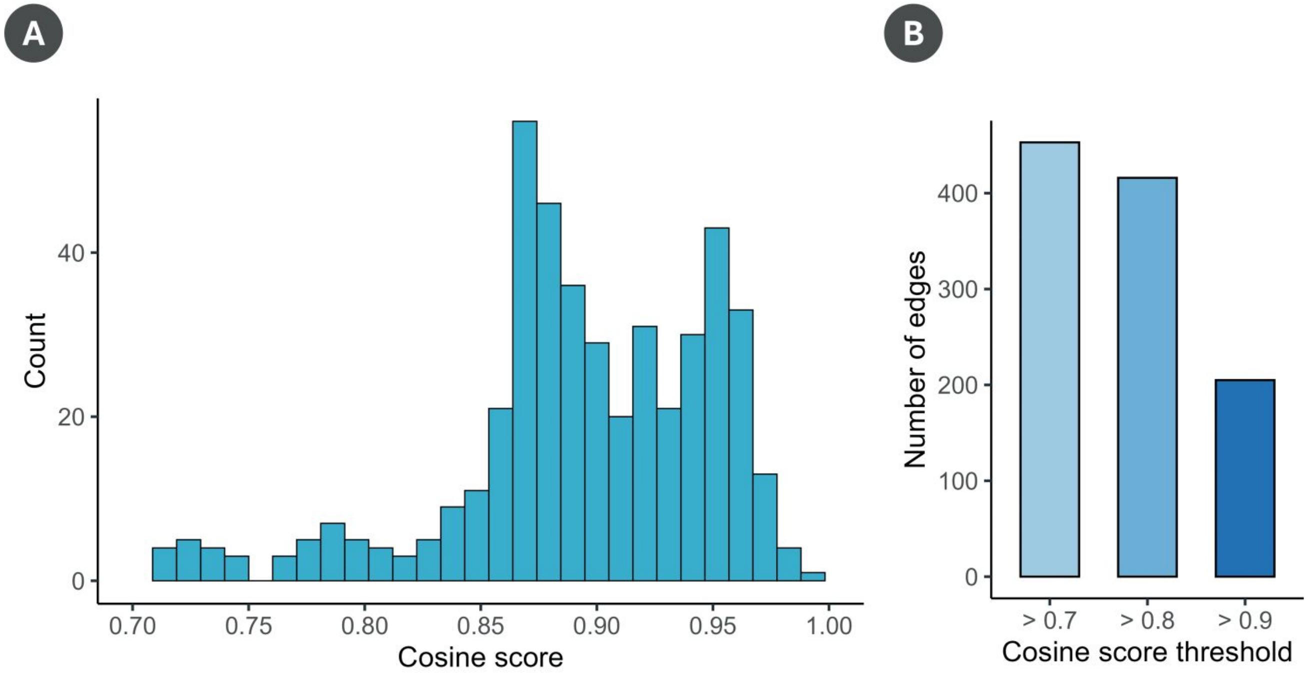

Figure S13. A) Histogram showing the distribution of pairwise cosine similarity values and the corresponding number of nodes. **B)** Bar chart showing the total number of edges at different pairwise cosine similarity thresholds from the overall molecular network analysis presented in Figure 4 of the manuscript.

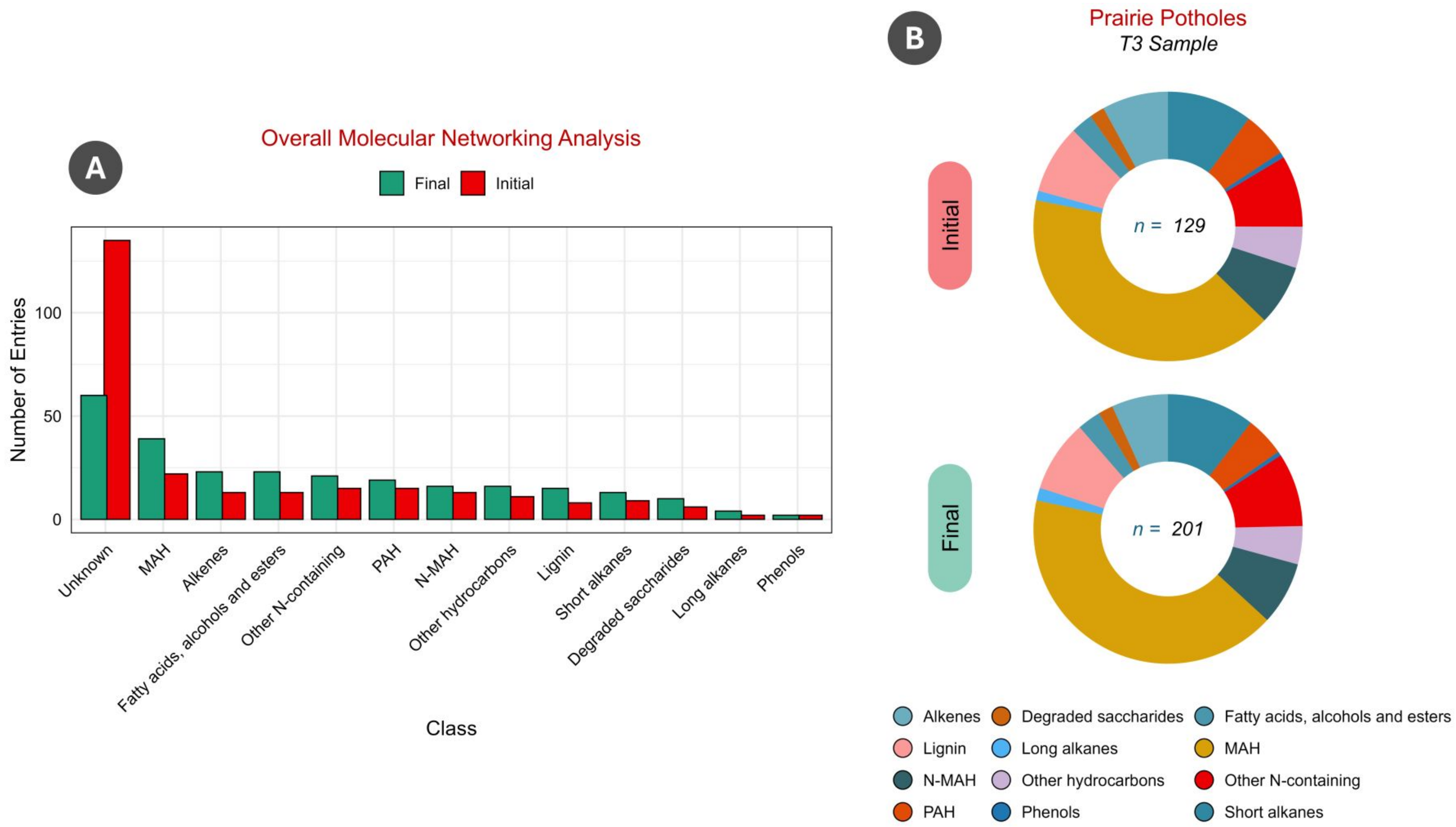

Figure S14. A) Changes in the number of annotated nodes within each compound class in overall molecular networking analysis (as presented in Figure 4 of main text) based on Python workflow. 'Unknown' refers to the unclassified nodes. **B)** Compound class distribution of prairie pothole sample (T3) before and after implementation of the Python workflow. *n* inside each donut represents the total number of classified compounds.

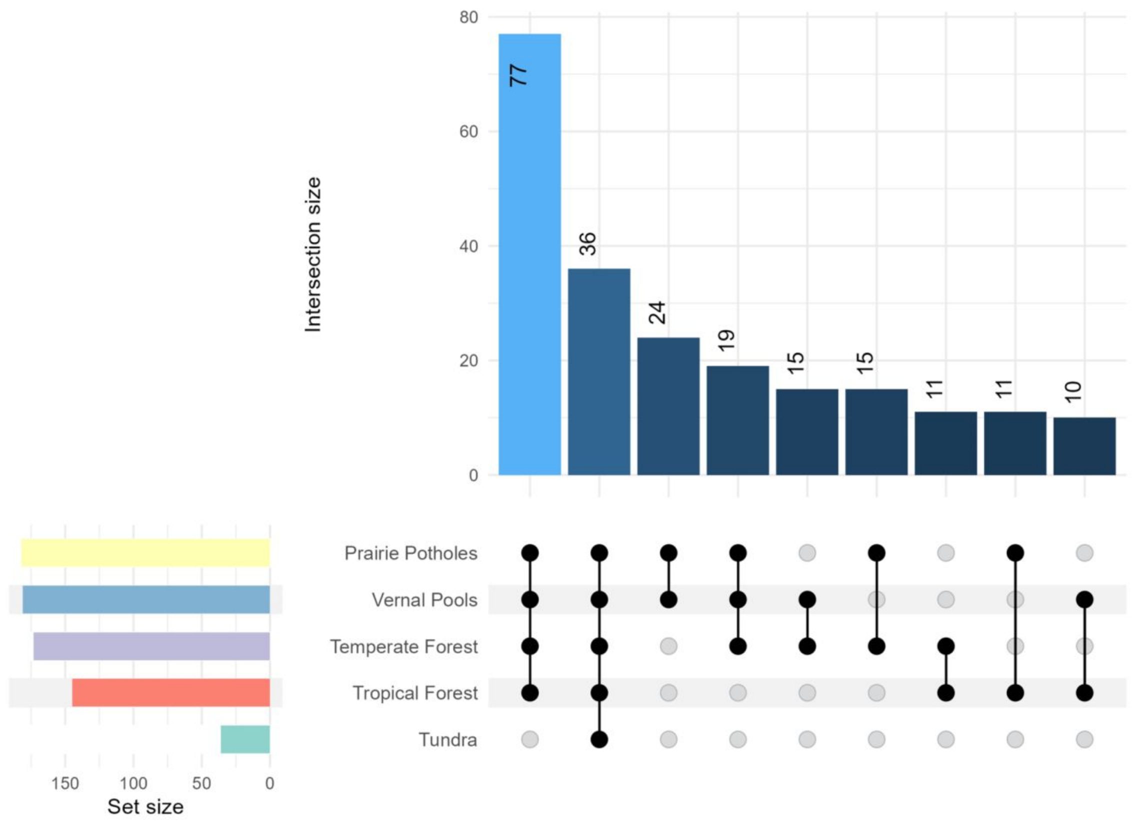

Figure S15. Quantitative representation of compounds across ecosystems derived from the overall molecular networking analysis (Figure 4, main text). Horizontal bars on the left indicate the number of nodes associated with each ecosystem, while vertical bars (shown in different shades of blue) depict the total number of nodes shared among various ecosystem combinations. Combinations occurring fewer than 10 times are omitted for clarity.

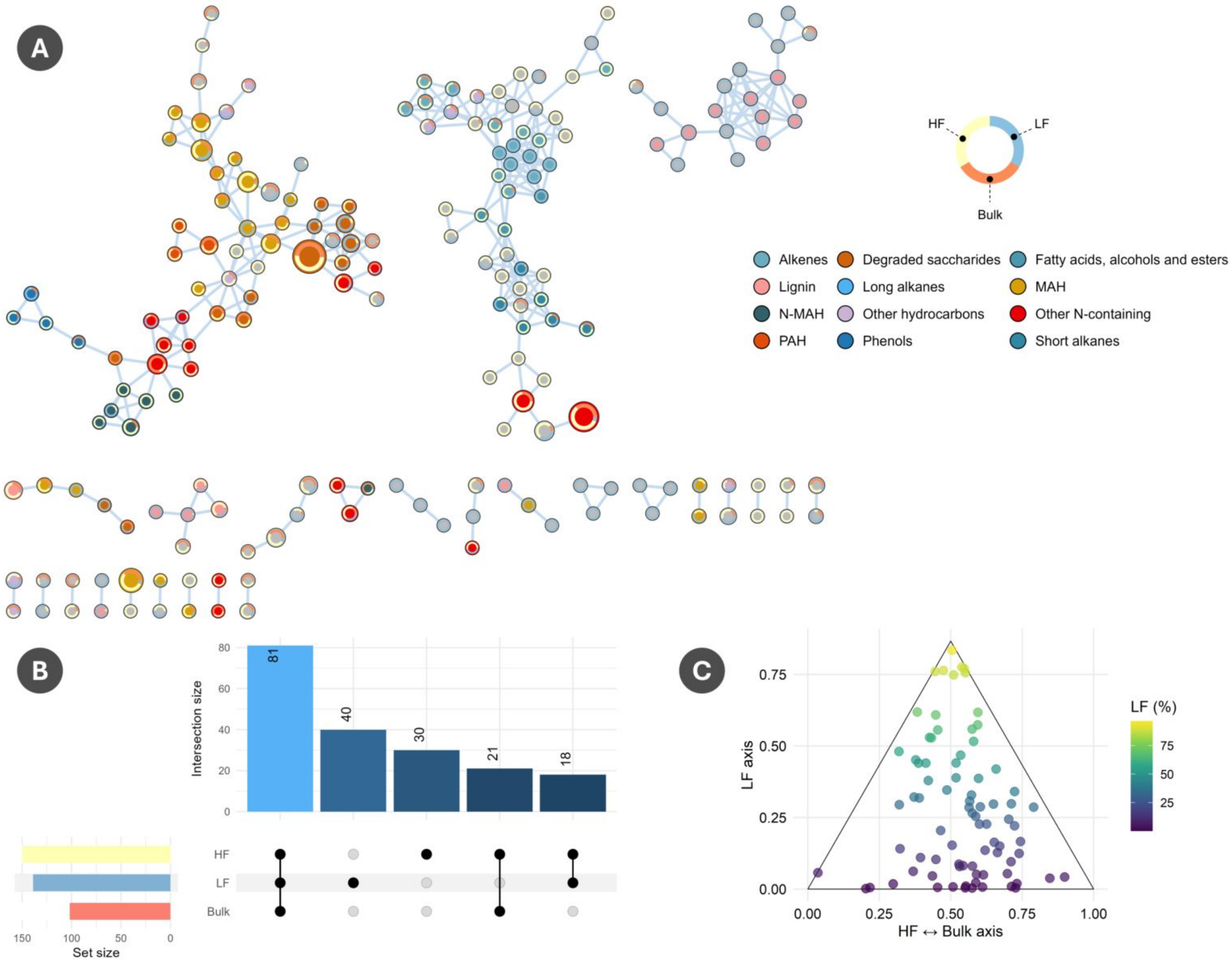

Figure S16. A) Molecular networking analysis of the bulk and two density fractions (heavy fraction, HF, and light fraction, LF) of grassland soil samples. **B)** quantitative representation of distribution of compounds in different density fractions. **C)** 2D ternary projection of the bulk, HF, and LF compositions. Each point represents a sample normalized to 100%. Position within the triangle reflects the relative proportions: left = Bulk-rich, right = HF-rich, top = LF-rich. The vertical axis and color shading both indicate LF contribution to overall composition.
